# Analysis of tumor environmental response and oncogenic pathway activation identifies distinct basal and luminal features in HER2-related breast tumor subtypes

**DOI:** 10.1186/bcr2899

**Published:** 2011-06-07

**Authors:** Michael L Gatza, Hsiu-Ni Kung, Kimberly L Blackwell, Mark W Dewhirst, Jeffrey R Marks, Jen-Tsan Chi

**Affiliations:** 1 Duke Institute for Genome Sciences and Policy, Duke University Medical Center, CIEMAS Building, 101Science Dr, Durham, NC 27708, USA; 2 Department of Molecular Genetics and Microbiology, Duke University, CARL Building, Research Dr., Durham, NC 27710, USA; 3 Department of Anatomy and Cell Biology, College of Medicine, National Taiwan University, Taipei, Taiwan; 4 Department of Medicine, Duke University Medical Center, 3818 Duke South, Durham, NC 27710, USA; 5 Department of Radiation Oncology, Duke University Medical Center, 201 MSRB, Research Dr., Durham, NC 27710, USA; 6 Department of Surgery, Duke University Medical Center, B216 LSRC, Durham, NC 27710, USA

## Abstract

**Introduction:**

Breast cancer heterogeneity occurs as a consequence of the dysregulation of numerous oncogenic pathways as well as many non-genetic factors, including tumor microenvironmental stresses such as hypoxia, lactic acidosis, and glucose deprivation. Although the importance of these non-genetic factors is well recognized, it is not clear how to integrate these factors within the genetic framework of cancer as the next logical step in understanding tumor heterogeneity.

**Methods:**

We report here the development of a series of gene expression signatures to measure the influences of microenvironmental stresses. The pathway activities of hypoxia, lactic acidosis, acidosis and glucose deprivation were investigated in a collection of 1,143 breast tumors, which have been separated into 17 breast tumor subgroups defined by their distinct patterns of oncogenic pathways. A validation dataset comprised of 547 breast tumors was also used to confirm the major findings, and representative breast cancer cell lines were utilized to validate *in silico *results and mechanistic studies.

**Results:**

Through the integrative pathway analysis of microenvironmental stresses and oncogenic events in breast tumors, we identified many known and novel correlations between these two sources of tumor heterogeneity. Focusing on differences between two human epidermal growth factor receptor 2 (HER2)-related subgroups, previously identified based on patterns of oncogenic pathway activity, we determined that these subgroups differ with regards to tumor microenvironmental signatures, including hypoxia. We further demonstrate that each of these subgroups have features consistent with basal and luminal breast tumors including patterns of oncogenic signaling pathways, expression of subtype specific genes, and cellular mechanisms that regulate the hypoxia response. Importantly, we also demonstrate that the correlated pattern of hypoxia-related gene expression and basal-associated gene expression are consistent across HER2-related tumors whether we analyze the tumors as a function of our pathway-based classification scheme, using the intrinsic gene list (ERBB2+), or based on HER2 IHC status. Our results demonstrate a cell lineage-specific phenomenon in which basal-like tumors, HER2-related tumors with high hypoxia, as well as normal basal epithelial cells express increased mRNA levels of *HIF-1α *compared to luminal types and silencing of HIF-1α results in decreased expression of hypoxia-induced genes.

**Conclusions:**

This study demonstrates differences in microenvironmental conditions in HER2-related subgroups defined by distinct oncogenic pathway activities, and provides a mechanistic explanation for differences in the observed hypoxia response between these subgroups. Collectively, these data demonstrate the potential of a pathway-based classification strategy as a framework to integrate genetic and non-genetic factors to investigate the basis of tumor heterogeneity.

## Introduction

Breast cancer is a collection of distinct diseases characterized by differences in oncogenic mechanisms and clinical characteristics including prognosis and response to therapeutic regimens. Clinically, tumors are classified on the basis of tumor size, visual characteristics, and a limited number of histochemical markers including estrogen receptor, progesterone receptor and HER2 receptor status. While the strongest and most consistent division in breast cancer is between the basal and luminal types and systematic differences strongly suggest a different cell of origin for these two dominant categories [1-5] additional molecular or mechanistic heterogeneity can be identified within classes.

Amplification of HER2 (*ERBB2*) occurs in approximately 15 to 25% of human breast tumors and defines a clinically unique subgroup of breast tumors [6]. Despite the introduction of Herceptin (trastuzumab), a monoclonal antibody that therapeutically targets HER2, the prognosis for these patients remains poor since a substantial number of patients either fail to respond to this therapy or develop resistance over time [7-10]. Therefore, it is clear that additional heterogeneity exists within this class of tumors. A number of recent studies have demonstrated the genomic complexity of HER2+ tumor heterogeneity, reporting that these tumors have varied and complex patterns of copy number alterations, global gene expression, and DNA methylation [11,12]. Moreover, molecular subtypes based on analysis of gene expression patterns [13] as well as more complex analyses of patterns of oncogenic and tumor suppressor pathway activity [14] have identified further subtypes that provide additional insight into the molecular and clinical heterogeneity of HER2+ tumors beyond the original ERBB2+ classification [1-5]. These studies also suggest that further investigation of the basis of HER2+ tumor heterogeneity, including the interaction of genetic and non-genetic factors, is necessary to fully elucidate the molecular mechanisms driving HER2-mediated oncogenesis.

Inherited germline mutations as well as acquired somatic mutations, unique to each individual patient, provide a source of variation at the genetic level. However, additional variation in the environmental conditions further influence tumor phenotypes and select for tumors adapted for growth within these microenvironments. The most visible of these environmental factors relate to common changes in the physical and chemical alterations of cancers, including reduced oxygen tension (hypoxia), high lactate, extracellular acidosis (lactic acidosis), and glucose depletion [15-18]. These changes may result from poor tissue perfusion, abnormal tumor vasculature, and/or genetic dysregulation of metabolism in the cancer cell.

Hypoxia (or low pO_2_) is recognized as a risk factor for poor clinical outcomes and increased metastasis, partly due to the gene expression response to hypoxia [18]. The cellular response to hypoxia is triggered by the stabilization of hypoxia regulators, HIF-1α or EPAS1 (HIF-2α) proteins, under limited oxygen. However, the HIF protein stabilization and hypoxia response are not limited to low pO_2 _and may also result from a wide range of genetic alterations and signaling malfunctions. These conditions represent "pseudo-hypoxia" and include the loss of *VHL *[19], *TP53 *[20], or *PTEN *[21] or activation of PI3-kinase/Akt [22] and HER2 [23] pathways. In addition, the *HIF *genes can also be expressed at different levels among different cells and contribute to the varying degrees of hypoxia response [24,25]. Hypoxia also favors glycolysis and leads to lactic acidosis with the accumulation of lactate and acidity in solid tumors. Avid glucose uptake and glycolysis can lead to glucose deprivation, another stress found in solid tumors [26,27]. Glucose deprivation triggers activation of AMPK and LKB-1, which in turn activates TSC1/TSC2 and inhibits the central energy sensor mTOR [28]. In addition, long term exposure to glucose deprivation has been shown to contribute to *KRAS *mutation in human cancers [29].

Given the importance of these microenvironmental stresses, many methods have been developed to measure their occurrence in solid tumors. These methods include direct measurements of oxygen tension (EF5, Eppendorf polarographic probe), acidity (pH probes), and lactate/glucose levels (bioluminescence technique) [30-33]. These approaches allow us to obtain a calibrated map of the respective metabolites' distributions within the tumor tissues that indicates relative abundance and spatial distribution. The level of each of these stresses in viable tumor areas shows large variations between tumors and can be related to tumor behavior and different clinical risks [30,34-38]. Therefore, an ability to assess these physiological parameters prior to treatment has the potential to aid in the development of novel therapeutic strategies for individual patients.

Drawbacks to these *in vivo *tumor measurements are that they are frequently invasive, technically challenging, or require tumors to be snap-frozen in a sophisticated laboratory setting; they are not amenable to routine and optimal clinical care. We have been working towards developing a conceptual framework to evaluate and incorporate the influences of these stresses to better understand the basis of tumor heterogeneity. The influence of tumor environment can be manifested by the expression of "endogenous" response genes; for example, tissue hypoxia can be inferred by elevated levels of *CA9 *[39]. When the cellular response genes are captured by microarrays in the form of "gene signatures", these *in vitro *derived gene expression signatures are capable of quantitatively assessing and classifying tumors based on their environmental profile analogous to oncogenic pathway activation and other phenotypes [24,40-45].

We recently reported the development of a tumor classification strategy based on patterns of oncogene and tumor suppressor activity as measured by gene expression signatures of each oncogenic pathway [14]. This study identified 17 breast tumor subgroups that are associated with the intrinsic subtypes of breast cancers and have shown an ability to further delineate potential mechanisms of disease by identifying novel patterns of oncogenic pathway activity and copy number variation between subgroups [1,46]. In the current study, we apply a similar framework towards understanding the nature of environmental and metabolic stresses in the context of defined lineage and oncogene specific tumor characteristics. Our findings indicate that a strong hypoxic response is the most characteristic of basal type tumors, which extends to a subset of HER2 related cancers that express characteristics consistent with derivation from the basal lineage. These differences in the hypoxia responses are likely due to the cell-type specific expression levels of HIF mRNAs which are associated with mammary differentiation programs.

## Materials and methods

### Gene expression signatures and tumor samples

The methods and a description of the training data used to develop the oncogenic pathway gene expression signatures have been published [14] and a detailed description of the methods, including the training data, for the development of the microenvironment signatures as well as for signatures to measure basal-luminal and subgroup 7/10 characteristics are provided in the Additional file 1, Supplemental methods section. Signature parameters, genes, and regression weights for the microenvironment signatures are reported in Additional file 2 Table S1. Briefly, a signature represents a group of genes that collectively demonstrate a consistent pattern of expression in relation to a given phenotype. Each signature is derived from the first principal component or the factor corresponding to the largest singular value as determined by singular value decomposition. A binary probability regression model is then estimated using Bayesian methods based on the vectors representing the two phenotypic states of the training data. The gene selection, identification, and regression model is based solely on the training data and maintains statistical independence from the validation dataset. This enables evaluation of predictive probabilities of each of the two phenotypic states in the training data for each sample in the validation datasets. Two previously described meta-datasets containing expression data from 1,143 and 547 human breast tumor samples respectively, were collected from 10 and 2 independent datasets for which Affymetrix HG-U133 CEL files were publicly available [14]. A summary of publicly available samples utilized in the study is provided in Additional file 3, Table S2. Samples were first RMA or MAS5 normalized using Affymetrix Expression Console ver1.0. MAS5 data were log2 transformed and both RMA and log2MAS5 datasets were filtered to include only those probes on the Affymetrix (Santa Clara, CA, USA) HG-U133A array. Samples were then normalized by Bayesian Factor Regression Analysis (BFRM) to remove technical variation as previously reported [14]. All studies using human data were performed in compliance with the Helsinki Declaration.

### Cell culture

AU565 and HCC202 cells were maintained in RPMI with 15% FBS and 1% penicillin/streptomycin while MCF7, BT474, CAMA1, BT20, MDAMB231, and MDAMB157 were cultured in DMEM with 10% FBS and 1% penicillin/streptomycin. Primary luminal and basal cells are obtained from human breast organoids and isolated as described [47]. For hypoxia, cells were cultured under 1% O_2 _for 4 or 24 hours.

### RNA interference and overexpression

The cells were plated in six-well plates at a density of 3 × 10^5 ^cells per well. For RNAi, cells were transfected with control non-targeting or siRNA against HIF-1α or HIF-2α with lipofectamine (Invitrogen, Carlsbad, CA, USA). RNA was extracted 48 hours after transfection and the levels of indicated transcripts were examined by real-time RT-PCR.

### Real-time RT-PCR

Total RNAs from cells, including cancer cell lines and primary breast cells, under normal culture condition or 48 h of hypoxia (1% O_2_) were isolated using TRIzol reagent (Invitrogen, Carlsbad, CA, USA) and then reverse-transcribed to cDNAs with SuperScript II reverse transcription kit (Invitrogen, Carlsbad, CA, USA) and used for real-time PCR with Power SYBRGreen PCR Mix (Applied Biosystem, Foster City, CA, USA) and primers for *HIF1A *(TGCTCATCAGTTGCCACTTC, CAGAAGTTTCCTCACACGCA), *EPAS1 *(CCATGTCTCCACCTTCAGA, GCTTCAGCTTCAGCTTGTTG), *ACTB (β-actin) *(CTCTTCCAGCCTTCCTTCCT, AGCACTGTGTTGGCGTACAG), *CA9 *(TCCTCAAGAACCCCAGAATAA, CCTCCATAGCGCCAATGACT), *GLUT1 *(AACTCTTCAGCCAGGGTCCAC, CACAGTGAAGATGATGAAGAC), *VEGFA *(TGCTCTACCTCCACCATGCCAA, TGATGATTCTGCCCTCCTCCTTC). The relative levels of each gene from real-time PCR were normalized with the level of *β-actin*.

### Analysis of gene expression levels

To compare relative levels of expression for individual genes or the average of several related genes, RMA normalized Affymetrix U133A expression data from the primary or the validation datasets were compared. The Affymetrix probes used for each gene or condition are reported in the Supplemental methods. For the analysis of the average basal-like or hypoxia-related gene expression, probes associated with each condition, reported in the Supplemental methods, were averaged across each sample. An unpaired t-test or Mann-Whitney U-test, as reported in the text, was used to compare between groups and a linear regression was used to compare expression values on a continuous variable scale.

## Results

### Analysis of co-regulation of oncogenic signaling and microenvironment pathways in breast cancer

**Figure 1:**
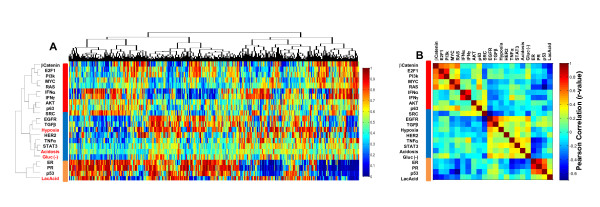
**Patterns of oncogene, tumor suppressor and microenvironment pathway activity in human breast cancer**. (**A**) Heatmap depicting the two-way hierarchical clustering of patterns of indicated microenvironment stresses (labeled in red) together with oncogenic pathway activities (labeled in black) in a collection of 1,143 human breast tumors. Red and blue indicates a high and low probability of pathway activity, respectively. (**B**) Pearson Correlation Coefficient (r) values between the activities of the 22 indicated pathways.

To investigate the influence of genetic and non-genetic factors in human breast cancer, we first constructed gene expression signatures to define the influences of a series of microenvironmental stresses and then analyzed them in the context of previously defined oncogenic pathways. We developed a series of novel gene expression signatures capable of quantitatively assessing the influence of hypoxia, lactic acidosis, acidosis, and glucose depletion (Glu(-)) using previously published gene expression studies of breast cells [42,44,48] (Additional file 4 Figure S1 and Additional file 3, Table S1). The pathway activities of these stress signatures were integrated with a series of 18 previously reported oncogenic pathway signatures using a Bayesian binary regression strategy to quantitatively assess the stress pathway activities in a collection of 1,143 human breast tumors that were collected from 10 independent datasets and normalized by Bayesian Factor Regression Modeling (BFRM) to remove technical variation [14]. The predicted hypoxia and lactic acidosis pathway activities were further validated by their respective significant correlations with the average expression levels of previously defined hypoxia-inducible genes [24] and the expression of *TXNIP*, which is induced by lactic acidosis and is the putative marker of the lactic acidosis response [44] (Additional file 4, Figure S1). Concurrently, the predicted probabilities of 18 oncogenic signaling pathways were determined in this dataset [14] (Figure 1) to enable the integrative analysis.

Two-way hierarchical clustering (Figure 1A) and a Pearson correlation (Figure 1B) was calculated to define the statistical correlation between co-activated pathways These analyses identify three separate clusters of pathways with a clear clustering among the hypoxia, Glu(-) and acidosis pathways together with the EGFR, TGFβ, HER2, TNFα and STAT3 pathways (Figure 1A). A significant relationship was also identified between the ER, PR, and p53 pathways which were found to be grouped with lactic acidosis pathway activity in a second cluster (Figure 1). Our previous study on the patterns of oncogenic pathway activity in human breast tumors [14] demonstrated clear correlations between the interferon pathways as well as between MYC and RAS, which are consistent with the findings of many other studies [49-51]. As illustrated in Figure 1A, these correlations persist with the inclusion of environmental conditions. Moreover, we are able to confirm the previously identified positive and negative relationships between lactic acidosis with p53 and PI3K/AKT pathways, respectively [42]. Finally, the positive correlation and clustering of the hypoxia pathway with EGFR, TGFβ and STAT3 was consistent with their reported reciprocal positive regulation [52,53]. The re-discovery of these connections among different pathways in human tumors provides a measure of validation for the expression signatures and the predictive approach to integrate genetic and non-genetic sources of tumor phenotypes.

### Analysis of microenvironment conditions in pathway-defined subgroups of breast cancer identifies differences between HER2 related subgroups

**Figure 2:**
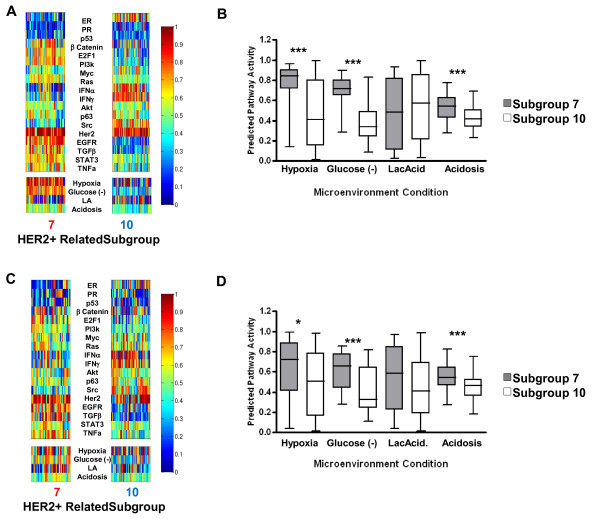
**HER2 related subgroup 7 is characterized by increased pathway activities of hypoxia and other stresses**. (**A**) Heatmap identifying patterns of oncogenic and micro-environmental pathways activity in the HER2 related tumor subgroups 7 (*n *= 53) and 10 (*n *= 59) in the primary dataset of 1,143 tumors; red indicates high predicted pathway activity, blue, low pathway activity. (**B**) Quantitative assessment of predicted probabilities of microenvironmental conditions in primary dataset demonstrating significantly higher hypoxia (*P *<0.0001), glucose deprivation (*P *<0.0001), and acidosis (*P *<0.0001) but no difference in lactic acidosis levels (*P *= 0.5140). (**C**) Heatmap identifying patterns of oncogenic and micro-environmental pathways activity in the HER2 related tumor subgroups 7 (*n *= 37) and 10 (*n *= 47) in the validation dataset of 547 tumors; red indicates high predicted pathway activity, blue, low pathway activity. (**D**) Quantitative assessment of predicted probabilities of microenvironmental conditions in primary dataset demonstrating significantly higher hypoxia (*P *= 0.0149), glucose deprivation (*P *<0.0001), and acidosis (*P *= 0.0008) but no difference in lactic acidosis levels (*P *= 0.0697).

We recently reported the identification of 17 breast cancer subgroups based on patterns of 18 different oncogene and tumor suppressor pathway activities [14]. These subgroups, while not simply a refinement of the molecular subtypes defined by the intrinsic gene list (that is, basal, luminal A, luminal B, ERBB2+, normal-like), have been shown to be significantly associated with these intrinsic subtypes to further delineate biological and clinical heterogeneity within these groups. Therefore, we next investigated microenvironmental stress pathway activities in the context of these 17 subgroups (Additional file 4, Figure S2). Interestingly, in subgroups 7 and 10, which are characterized by high levels of HER2 pathway activity (Additional file 4, Figure S2) and have been shown to be enriched for ERBB2+ tumors [14], the pathway activities of hypoxia/EGFR/TNFα/TGFβ/STAT3 showed consistently higher levels in subgroup 7 than subgroup 10 tumors (Figures 2A, 2B). As illustrated in Figure 2Aand quantitatively assessed in Figure 2B, clear differences in patterns of predicted microenvironmental pathway activities, specifically hypoxia response, glucose depletion, and acidosis, as well as a number of hypoxia-associated oncogenic signaling pathways, including EGFR/TNFα/TGFβ/STAT3, are characteristically different between subgroups 7 and 10. To quantitatively assess these differences, an unpaired t-test was used to compare patterns of predicted pathway activity in each subgroup (Figure 2B and Additional file 4, Figure S3). We found that subgroup 7 has significantly (*P *<0.0001, unpaired t-test) higher levels of predicted hypoxia response and many other pathways that were shown to be clustered with hypoxia, including EGFR, TGFβ, TNFα, STAT3, Glu(-) and acidosis (Figure 2A and Additional file 4 Figure S3). In contrast, tumors assigned to subgroup 10 had significantly higher levels of ER, PR, IFNα, IFNγand SRC pathway activity (Additional file 4, Figure S3). In order to validate the differences in patterns of microenvironmental pathway activity, a validation dataset comprised of 547 independent breast tumor samples was assessed for patterns of oncogenic pathway activity and assigned to subgroups [14]. Patterns of microenvironmental pathway activity were then assessed (Figure 2C) and examined quantitatively (Figure 2D) demonstrating patterns of microenvironmental pathway activity in subgroups 7 and 10 consistent with the discovery dataset of 1,143 samples. Specifically, this independent, validation dataset also demonstrates a significant difference (unpaired t-test) in patterns of hypoxia response, glucose depletion, and acidosis (Figure 2D).

### Subgroup 7 tumors exhibit a strong hypoxia response

**Figure 3:**
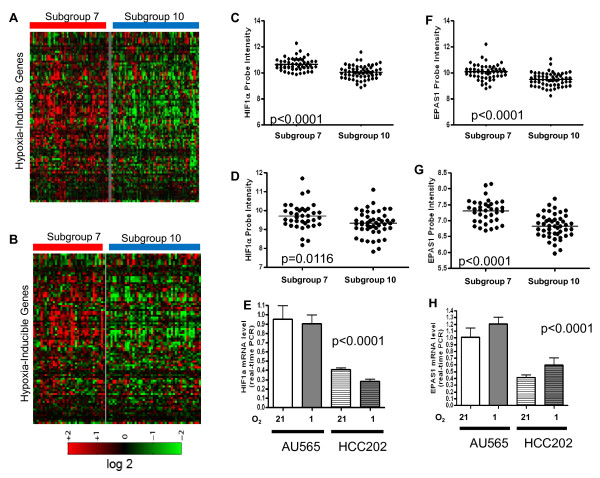
**Subgroup 7 tumors are characterized by increased levels of hypoxia-related gene expression**. Subgroup 7 tumors express higher levels of hypoxia-induced genes as compared to subgroup 10 tumors in the (**A**) primary and the (**B**) validation datasets. Subgroup 7 tumors express significantly higher levels of *HIF-1α *mRNA in the (**C**) primary dataset (*P *<0.0001) and (**D**) the validation dataset (*P *= 0.0016) when compared to subgroup 10 tumors as examined by microarray analysis. (**E**) Subgroup 7 representative cell line AU565 (*P *<0.0001) demonstrated higher levels of *HIF-1α *expression as compared subgroup 10 cell line, HCC202 when examined by RT-PCR under normal (21% O_2_) and hypoxic (1% O_2_) conditions. Similarly, significantly higher expression of EPAS1 (HIF-2α) was noted in the subgroup 7 in (**F**) the primary dataset (*P *<0.0001) and (**G**) the validation dataset (*P *<0.0001) when compared to subgroup 10 tumors by microarray analysis. AU565 (*P *<0.0001) demonstrated higher levels of *EPAS1 (HIF2α) *expression as compared subgroup 10 cell line, HCC202, when examined by RT-PCR under normal (21% O_2_) and hypoxic (1% O_2_) conditions.

Since tumors in subgroup 7 were characterized by a higher level of hypoxia response (Figure 2 and Additional file 4, Figure S3) when compared to subgroup 10 tumors in both a primary and validation dataset, we next examined whether these tumors showed differences in expression of known hypoxia-induced genes. As shown in Figure 3A and 3B (and in higher resolution in Additional file 4, Figures S4A and S4B), subgroup 7 tumors demonstrated higher expression levels of a panel of previously-defined common hypoxia-induced genes [24] (list of 89 Affymetrix probes provided in Supplemental methods) in both the primary (Figure 3A) and validation (Figure 3B) datasets. Consistent with these observations, significantly higher mRNA levels of many hypoxia-induced genes including *VEGFA (P *= 0.0004, Mann-Whitney *U*-test), *EGLN3 (P *<0.0001), *GLUT1 (P *= 0.0121), and *DEC1 (P *<0.0001) were observed in subgro up 7 tumors (Additional file 4, Figure S5). Collectively these data suggest that tumors in subgroups 7 and 10, while both sharing high HER2 pathway activity, differ not only in patterns of oncogenic pathway signaling but are also characterized by differences in the degrees of hypoxia response.

In order to understand the basis for the exaggerated hypoxia response observed in subgroup 7 tumors, we next analyzed gene expression levels of hypoxia regulators in these tumors. It was determined that subgroup 7 tumors demonstrated a greater than two-fold higher level of mRNA expression (*P *<0.0001, Mann-Whitney *U*-test) for both *HIF1A (HIF-1α*) (Figures 3C, D) and *EPAS1 (HIF-2α) *(Figures 3F, G) as compared to subgroup 10 tumors in both the primary (Figures 3C, F) and validation datasets (Figures 3D, G). While HIF-1α and HIF-2α are usually regulated at the protein level via oxygen-dependent degradation, several studies have reported a similar mRNA induction associated with a stronger hypoxia response [24,25]. To further validate the hypoxia response in subgroup 7 tumors, a number of additional known hypoxia regulators were identified from BioCarta, and it was determined that subgroup 7 tumors exhibited significantly higher levels of expression of *ARNT (P *= 0.0078), *EP300 (P *= 0.009), *JUN (P *<0.0001), and *LDHA (P *= 0.0092) (Additional file 4, Figure S6). Because several studies have also linked the level of HER2 amplification to the hypoxia pathway [23,54], we compared the mRNA levels of HER2 among these two HER2 related subgroups and found no significant difference in either the primary or validation datasets (Additional file 4, Figure S6).

As we noted previously [14], a clear advantage of utilizing the pathway-based classification scheme is an ability to assign new samples including cancer cell lines to corresponding subgroups based on the similarity of pathway composition. Therefore, we took advantage of this ability to identify *in vitro *cell culture model systems to test differences between subgroups 7 and 10. Using a previously analyzed dataset of 50 breast cancer cell lines [14], we identified two HER2+ breast cancer cell lines representative of subgroup 7 (AU565) and subgroup 10 (HCC202), each with a probability of subgroup membership greater than 0.90. Consistent with tumor data, we determined that the subgroup 7 cell line (AU565) has significantly higher mRNA levels of *HIF1A (HIF-1α*) (Figure 3E) and *EPAS1 (HIF-2α*) (Figure 3H) when compared to the subgroup 10 cell line HCC202. Moreover, when both cell lines were grown under hypoxic conditions, AU565 cells exhibited a much stronger hypoxia response as measured by higher mRNA levels of several hypoxia-inducible genes (Additional file 4, Figure S7). This is consistent with an enhanced hypoxia response and higher hypoxia-inducible factor levels in the subgroup 7 breast tumors.

### HER2 related subtypes exhibit basal and luminal type cell features

It has been reported that basal-like breast tumors have a significantly more robust hypoxia response compared to luminal-type breast cancers [55], similar to the differential hypoxia response between the two identified HER2 related subgroups. In addition, basal breast tumors are characterized by low ER and PR expression as well as high levels of *EGFR *[1,46], elevated expression of *AKT3 *[56], *CD44 *and *MET *[57] and decreased *GATA3 *expression [1]; features that are also consistent with each of the HER2 related subgroups investigated in the current study. Recent studies have also suggested that HER2 related tumors may be derived from late luminal progenitor cells, an intermediate differentiation stage between luminal progenitor cells from which basal-like tumors arise and differentiated luminal cells from which luminal A and B tumors arise [58,59]. Therefore, we next investigated whether the two HER2 related subgroups might have additional characteristics consistent with basal and luminal tumors in addition to being HER2+.

**Figure 4:**
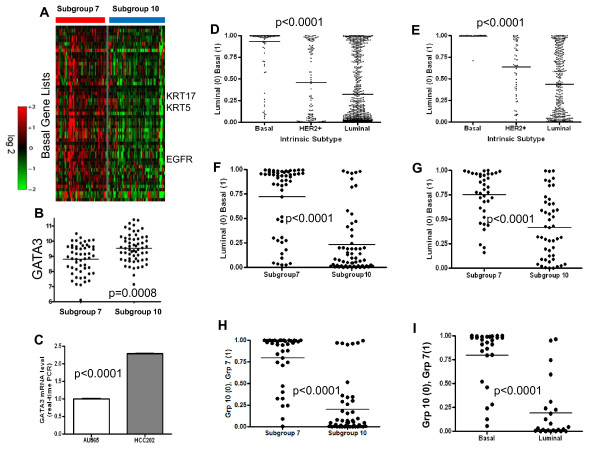
**HER2 related tumor subtypes are characterized by basal and luminal features**. (**A**) Heatmap showing the expression level of basal-type specific genes in subgroups 7 and 10 tumors. (**B**) Subgroup 10 tumors show significantly higher *GATA3 *mRNA expression (*P *= 0.0008) as compared to subgroup 7 tumors. (**C**) Subgroup 10 cell lines (HCC202) demonstrate higher levels of *GATA3 *expression than subgroup7 cell lines (AU565). (**D**) Validation of the luminal-basal signatures demonstrates an accurate prediction of basal (mean predicted probability: 0.933) and luminal (mean predicted probability: 0.321) breast tumors (*P *<0.0001). (**E**) Validation of luminal-basal signature in validation dataset shown accurate prediction of basal (mean predicted probability: 0.992) and luminal (mean predicted probability: 0.438) tumor status. (**F**) HER2 related tumors from the primary dataset of 1,143 samples in subgroup 7 demonstrate basal-like features while subgroup 10 tumors are associated with luminal-like features (*P *<0.0001). (**G**) HER2 related tumors in the validation dataset show significant enrichment of basal and luminal features in subgroups 7 and 10, respectively (*P *<0.0001). (**H**) Validation of subgroup 7/10 signature to predict subgroup7 and 10 characteristics of tumors assigned to subgroup 7 (mean predicted probability: 0.7950) and subgroup 10 (mean predicted probability: 0.2022) with a congruency rate of 84.5% (*P *<0.0001). (**I**) Classification of basal and luminal breast cancer cell lines demonstrates a congruency of 72% with basal and luminal status (*P *<0.0001).

We first measured differences in expression patterns of pathways and genes known to be differentially expressed in basal and luminal breast tumors. As shown in Figure 2 (and Additional file 4, Figure S3), tumors in subgroup 7 exhibit significantly higher hypoxia and EGFR pathway activity and lower levels of ER and PR pathway activity [55,60] when compared to tumors in subgroup 10, suggesting that tumors in subgroup 7 and 10 tumors, in addition to being HER2+, have basal-like and luminal-like patterns of pathway activities, respectively. Next, we compared the expression of several basal and luminal specific genes in the two HER2 related subgroups. As shown in Figure 4 (and validated in Additional file 4, Figure S8), subtype 7 tumors have significantly higher expression of basal related genes (Supplemental methods) including *AKT3, MET, EGFR, CD44 *and basal type cytokeratins (*KRT5 *and *KRT17*) (Figure 4A, and Additional file 4, Figures S8, S9, S10). In contrast, subgroup 10 tumors have higher expression of the luminal-specific master regulator *GATA3 *(Figure 4B) [61,62]. Consistent with this observation, HCC202 cells (subgroup 10) had significantly higher mRNA levels of *GATA3 *(Figure 4C) than AU565 cells (subgroup 7). Although these results demonstrate that subgroup 10 tumors do not express the majority of basal-type markers and do exhibit certain luminal-type features, these tumors did not have higher levels of luminal-type cytokeratins (*KRT18 *and *KRT19*) or several other luminal-specific markers (Additional file 4, Figure S11).

The differential expression of a number of luminal and basal-specific genes and pathways in subgroups 7 and 10 prompted us to further investigate this possibility at the genome-wide level. We used the expression of a well-annotated dataset of 24 luminal and 24 basal breast cancer cell lines to develop a gene expression signature with the ability to differentiate between basal and luminal tumor characteristics (Additional file 4, Figure S12A). The basal-luminal signature was first validated using a leave-one-out cross-validation among the cancer cell lines (Additional file 4, Figure S12B). The accuracy of the signature was then investigated by predicting the basal or luminal characteristics of tumors and then comparing the probability of basal or luminal status against the intrinsic subtype based on previously reported classifications of tumors [14] in both the primary and validation datasets. Our analysis determined that most basal tumors were strongly predicted to be basal-like (mean predicted probability: 0.933 and 0.992, primary and validation datasets, respectively). The majority of luminal tumors were predicted to have luminal-like characteristics (mean predicted probability: 0.321 and 0.438, respectively), albeit with a less uniform distribution of predicted probabilities as compared to basal tumors, likely owing to the high degree of heterogeneity in luminal breast tumors. However, the difference in the predicted probability between the intrinsic subtypes was still highly significant for both the primary and validation datasets (*P *<0.0001) (Figure 4D, 4E). In contrast to basal and luminal tumors, ERBB2+ tumors showed a wide-range of predicted basal-luminal characteristics (Figure 4D, 4E). However, when we segregated these tumors into the two pathway-based HER2 related subgroups (subgroups 7 and 10), we found that subgroup 10 tumors were predicted to be predominantly luminal in both the primary (congruence rate: 83%) and validation (congruence rate: 60%) datasets, while most tumors in subgroup 7 were predicted to be basal in the primary (congruence rate: 72%) and validation (congruence rate: 81.1%) datasets when a predicted probability of 0.5 is used as the cut-off for class assignment (Figure 4F, 4G) and to determine the level of agreement between 7/10 subgroup identity and basal/luminal characteristics (congruence rate).

To further examine the similarity between characteristics of each of the two HER2 related subgroups and basal/luminal cancer cell lines, we also performed the reciprocal analysis. We developed a gene expression signature, using the samples from the primary dataset as the training data, to differentiate between subgroup 7 and 10 tumor characteristics (Additional file 4, Figure S13). Among the genes identified in this signature, we noted, consistent with our earlier analyses, *GATA3 *and *MET *enrichment in subgroup 10 and 7 tumors, respectively, both of which are known to be preferentially expressed in luminal and basal tumor, respectively (Additional file 4, Figure S13). Although the intent of this signature is not to assign samples to subgroups 7 and 10, but instead to investigate differences in group characteristics, we first validated the ability of this signature to identify subgroup 7 and 10 characteristics using samples in the validation dataset. As illustrated in Figure 4H, even with the inclusion of samples with a lower probability (<0.70) of subgroup assignment, we demonstrate that the majority of tumors assigned to subgroups 7 were predicted to have subgroup 7-like characteristics (mean predicted probability: 0.7950) while samples assigned to subgroup 10 based on patterns of pathway activity were determined to have subgroup 10-like characteristics (mean predicted probability: 0.2022) with an overall congruence rate of 84.5% (*P *<0.0001). This signature and training model were then used to investigate the subgroup 7 and subgroup 10 characteristics of the breast cancer cell lines with established basal and luminal status. We found that the majority of the luminal and basal cancer cells were characteristic of subgroups 10 and 7, respectively (congruence rate 81.3%, *P *<0.0001), when a cutoff of 0.5 was used for class assignment and to calculate congruence rate (Figure 4I). Taken together, these data strongly suggested that subgroup 10 and subgroup 7 tumors have molecular characteristics that are consistent with luminal and basal type breast cancers, respectively. These results, consistent with several previous studies [3,11,63], suggest that HER2 related tumor heterogeneity may arise as a consequence of basal and luminal cell features and provide evidence supporting the hypothesis that the cell of origin for subgroup 7 tumors may have basal-like features, whereas subgroup10 tumors may arise from progenitor cells that have further differentiated and have features more consistent with luminal-like tumors.

### HER2 related tumors demonstrate significant positive correlations between hypoxia response, basal gene expression, and HIF gene expression

**Figure 5:**
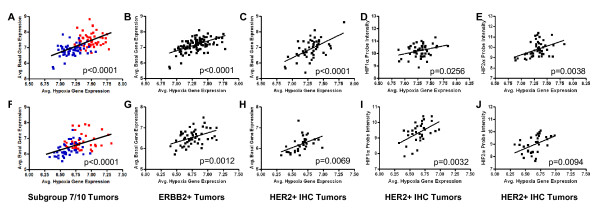
**HER2 related tumors demonstrate a significant correlative relationship between basal-like and hypoxia-related gene expression**. A positive correlation between basal-like and hypoxia-related gene expression was observed in the tumors assigned to subgroups 7 (highlighted in red) and 10 (highlighted in blue) in the (**A**) primary (*n *= 112, *P *<0.0001) and (**F**) validation (*n *= 84, *P *<0.0001) datasets; in tumors classified as ERBB2+ by the intrinsic gene list in the (**B**) primary (*n *= 115, *P *<0.0001) and (**G**) validation (*n *= 54, *P *= 0.0012) datasets; and in tumors that are HER2+ by IHC in the (**C**) primary (*n *= 49, *P *<0.0001) and (**H**) validation (*n *= 33, *P *= 0.0069) datasets. *HIF-1α *mRNA expression in IHC HER2+ tumors correlates with hypoxia-related gene expression in the (**D**) primary (*n *= 49, *P *= 0.0256) and (**I**) validation (*n *= 33, *P *= 0.0032) datasets. *EPAS1 (HIF-2α) *mRNA expression in IHC HER2+ tumors correlates with hypoxia-related gene expression in the (**E**) primary (*n *= 49, *P *= 0.0038) and (**J**) validation (*n *= 33, *P *= 0.0094) datasets.

Given that our results demonstrate a clear correlation among basal-like features, hypoxia response and HIF gene expression when comparing tumors in subgroups 7 and 10, we next investigated whether these observations can be further extended to the heterogeneity among HER2 related tumors irrespective of the classification strategy. Since different classification strategies lead to consistent but imperfect agreements sensitive to varying thresholds, we sought to further determine the relationship between the continuous variables of these properties. To investigate these correlations, we first compared the average expression of basal associated genes (detailed in Figure 4A) with the average expression of hypoxia-related genes (detailed in Figure 3A). As expected from our previous results, a significant, positive relationship was found to exist between average hypoxia-and basal-related gene expression in tumors assigned to subgroups 7 and 10 in both the primary (Figure 5A, P <0.0001) and validation (Figure 5F, P <0.0001) datasets. In order to investigate this relationship among HER2 related tumors, we next examined tumors classified as HER2 related (or ERBB2+) by the intrinsic gene list [61,62] (Figure 5B, G) as well as in those tumors that were reported to be HER2+ by IHC (Figure 5C, H). As illustrated in Figures 5B (*P *<0.0001) and 5C (*P *<0.0001) for the primary dataset, and validated in Figures 5G (*P *= 0.0012) and 5H (*P *= 0.0069), this relationship is evident regardless of which of the three independent methods is used to classify tumors as HER2+ or HER2 related.

Furthermore, our data suggest that differential tumor hypoxia response is associated in subgroups 7 and 10 tumors with expression of HIF mRNA (Figure 3). Among the tumors classified as HER2+ by IHC, there is a significant correlation between both *HIF1A (HIF-1α*) (Figure 5D, P = 0.0256) and *EPAS1 (HIF-2α*) (Figure 5E, P = 0.0038) expression and hypoxia-related gene expression. These results are further confirmed in the validation dataset as illustrated in Figures 5I (*P *= 0.0032) and 5J (*P *= 0.0094). Collectively, these data suggest that patterns of basal-related gene expression, as well as HIF levels, directly correlate with the expression of hypoxia-related genes in HER2 related tumors regardless of whether tumors are defined as HER2 related by a HER2 gene expression signature (subgroups 7/10), by the intrinsic gene list (ERBB2+), or by HER2 IHC status.

### Basal-like breast tumors exhibit an exaggerated hypoxia response and increased *HIF-1α *expression but not lower pO_2_

**Figure 6:**
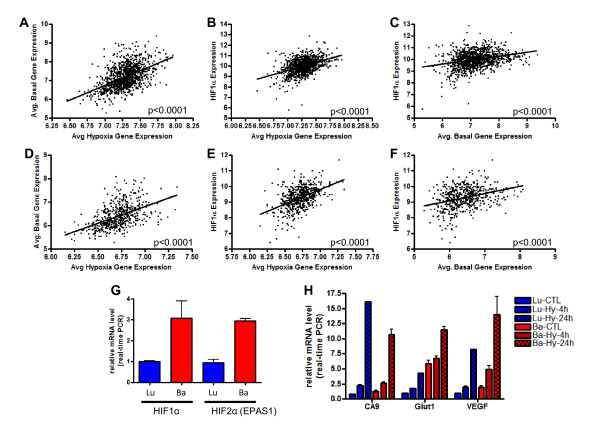
**Breast tumors with basal-like gene expression and basal epithelial cells differentially express hypoxia-related genes**. A significant positive correlation between basal-like and hypoxia-related gene expression was observed in breast tumors in the (**A**) primary (*n *= 1,143, *P *<0.0001) and (**D**) validation (*n *= 547, *P *<0.0001) datasets. A significant positive relationship was observed between HIF-1α gene expression and hypoxia-related gene expression in the (**B**) primary (*n *= 1,143, *P *<0.0001) and (**E**) validation (*n *= 547, *P *<0.0001) datasets. A significant positive relationship was observed between *HIF-1α *gene expression and basal-like gene expression in the (**C**) primary (*n *= 1,143, *P *<0.0001) and (**F**) validation (*n *= 547, *P *<0.0001) datasets. (**G**) Normal basal epithelial cells express higher mRNA levels of *HIF-1α (P *<0.0001) and *EPAS1 (HIF-2α) (P *<0.0001) as compared to normal breast luminal cells. (**H**) Normal basal epithelial cells demonstrate higher mRNA levels of three hypoxia-induced genes (*CA9, Glut1*, and *VEGFA*) under normal and hypoxic conditions as compared to normal luminal epithelia.

Our data suggest that tumors assigned to subgroup 7, which express high levels of *HIF-1α *and hypoxia-related genes, and subgroup 10, which express lower levels of *HIF-1α *and hypoxia-related genes, have characteristics in common with basal and luminal breast tumors, respectively. Since our results demonstrate that the correlation between average basal gene expression and average hypoxia-related gene expression is consistent across HER2 related tumors irrespective of classification strategy, we next investigated whether these relationships were evident across all breast tumors given that basal-like tumors have been previously reported to be characterized by an enhanced hypoxia response [55,64]. As illustrated in Figure 6, a direct correlation was observed in both the primary (Figure 6A, P <0.0001) and validation (Figure 6D, P <0.0001) datasets between basal-like and hypoxia-related gene expression. We further examined the correlation between *HIF-1α *expression and both hypoxia-related gene expression (Figure 6B, E) and basal-like gene expression (Figure 6C, F) across both the primary and validation dataset and demonstrated a significant (*P *<0.0001) relationship between both patterns of gene expression. These results, consistent with previous studies [55,64] collectively suggest that as tumors express more basal-like features, irrespective of HER2 status, they also demonstrate higher levels of hypoxia-related gene expression as well as higher levels of *HIF-1α*. Consistent with the latter point, we demonstrate that tumors assigned to subgroups 7 or that are classified as basal using the intrinsic gene list express higher levels of *HIF-1α *as compared to tumors assigned to subgroup 10 or classified as luminal, respectively (Additional file 4, Figure S14, *P *<0.0001).

Given that significantly higher mRNA levels of *HIF-1α *expression were noted in both HER2 related tumors and basal tumors with high levels of predicted hypoxia response, we next investigated whether the observed levels of predicted hypoxia response correlated with physical levels of oxygenation (pO_2_). We took advantage of data from our previously published study that measured pO_2 _levels *in situ *in human breast cancer using an Eppendorf electrode [33]. As shown in Figure S15, basal tumors in this cohort also showed significantly higher levels of hypoxia-induced gene expression (*P *= 0.0065) (Additional file 4, Figure. S15A) and *HIF-1α *mRNA (*P *= 0.0261) (Additional file 4 Figure S15B) when compared to luminal tumors. Importantly, no significant difference (*P *= 0.28) was observed for pO_2 _levels (Additional file 4, Figure S15C). Therefore, these data demonstrate that the exaggerated hypoxia response observed in basal-like tumors cannot be solely attributed to decreased oxygen levels. Instead, we postulated that intrinsically higher mRNA expression of hypoxia regulators, including *HIF-1α*, was responsible for the exaggerated hypoxia response in basal-type tumors.

Since basal-like breast tumors and HER2 related tumors with basal-like characteristics, including subgroup 7 tumors, demonstrated increased levels of *HIF-1α*, we investigated whether such differences are a cell-lineage phenomenon which can also delineate normal basal and luminal mammary epithelial cells. Primary breast epithelial cells were separated based on surface expression of EPCAM (TACSTD1) from reduction mammoplasty specimens and gene expression levels were determined by microarray [47]. Both arrays and real-time PCR showed that basal epithelial cells had a greater than three-fold higher level of both *HIF-1α *and *HIF-2α *expression compared to isogenic luminal epithelial cells (Figure 6G). When placed under hypoxic conditions, primary basal epithelial cells also demonstrated a significantly greater hypoxia response (Figure 6H). From these data, we hypothesize that the stronger hypoxia response is associated with higher expression of hypoxia regulators shared by both basal-like and HER2 related tumors with basal-like characteristics, including subgroup 7 tumors. Given that normal basal epithelial cells also demonstrate enhanced *HIF-1α *expression, the association may be a cell-lineage distinction that can be traced to systematic differences in the composition of hypoxia regulators between normal, non-transformed basal and luminal breast epithelial cells.

### *HIF-1α *mRNA is responsible for the strong hypoxia response in basal cells

**Figure 7:**
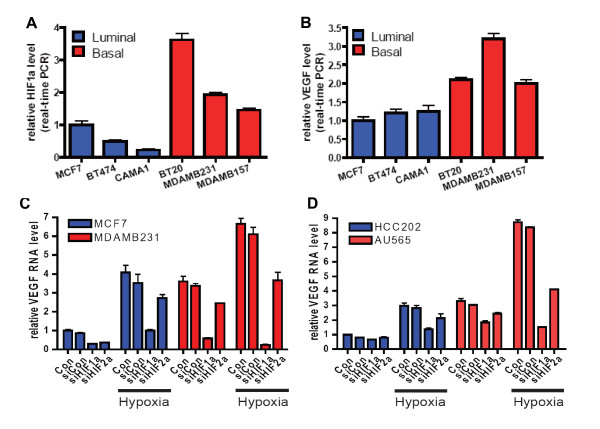
**HIF-1α silencing inhibits the exaggerated hypoxia response in basal and subgroup 7 cancer cell lines**. (**A**) Basal breast cancer cell line demonstrate higher *HIF-1α *mRNA levels when compared to luminal breast cancer cell lines (**B**) Basal breast cancer cell line demonstrate higher *VEGF *mRNA levels when compared to luminal breast cancer cell lines. (**C, D**) The silencing of *HIF-1α *but not *EPAS1 (HIF-2α *mRNA by siRNA inhibits hypoxia-induced gene expression (*VEGF*) levels in basal breast cancer cell lines (C) and HER2-basal subtypes cell lines (D) under both normal and hypoxic conditions as compared to their respective luminal-type breast cancer cell lines.

Since our results demonstrated a correlation between predicted hypoxia response and *HIF-1α *mRNA expression in both basal breast tumors and HER2 related tumors with basal-like characteristics, including subgroup 7 tumors, we next investigated whether *HIF-1α *mRNA expression directly regulated the exaggerated hypoxia-induced gene expression in these cells. We analyzed *HIF1A (HIF-1α *expression in six common breast cancer cell lines to determine whether basal breast cell lines, which have been previously reported to have a higher hypoxia response [64], also express higher levels of *HIF1A (HIF-1α) *and *EPAS1 (HIF-2α) *mRNA as compared to luminal breast cancer cell lines. We determined that the basal breast cancer cell lines (BT20, MDA-MB231, MDA-MB157) had consistently higher mRNA levels of both *HIF1A (HIF-1α) *(Figure 7A) and *EPAS1 (HIF-2α) *(Additional file 4, Figure S16A) when compared to the luminal cell lines (MCF7, BT474, CAMA1). Furthermore, we found that the basal breast cancer cell lines also exhibited elevated expression levels of three hypoxia-inducible genes (*VEGF, GLUT1*, and *CA9*) even under normoxia (Figure 7B and Additional file 4, Figure S16B, C). These results are consistent with previous studies reporting a strong constitutive hypoxia response and glycolytic phenotype in basal breast cancer cell lines [64,65].

To investigate the roles of *HIF1A (HIF-1α) *and *EPAS1 (HIF-2α) *in the hypoxia response, we reduced expression of both genes by siRNA-mediated gene silencing in MCF7 (luminal) and MDA-MB231 (basal) cells. This gene silencing significantly reduced both transcripts in MDA-MB231 cells to a level comparable to those in MCF7 cells (Additional file 4, Figure S17A, B). Silencing of *HIF-1α*, but not *HIF-2α*, mRNA significantly reduced the exaggerated induction of *VEGF *(Figure 7C), *GLUT1*, and *CA9 *expression (Additional file 4, Figure S17C, D) in MDA-MB231 cells under hypoxia (1% pO2). Importantly, the silencing of *HIF1A (HIF-1α)*, but not *EPAS1 (HIF-2α)*, in MDA-MB231 reduced the induction of these hypoxia-inducible genes to the levels comparable to MCF7 cells (Figure 7C and Additional file 4, Figure S17). Finally, we examined whether a similar mechanism may be also important for the stronger hypoxia response in the breast cancer cell lines in subgroup 7. Consistent with our findings in the basal-breast cancer cell line, AU565 cells (subgroup 7) demonstrated that silencing of *HIF1A (HIF-1α)*, but not *EPAS1 (HIF-2α)*, led to the abolishment of the exaggerated hypoxia response seen in these cells (Figure 7D and Additional file 4, Figure S18). Taken together, these data are consistent with the role of high levels of *HIF-1α *regulating the exaggerated hypoxia response in both basal-like cancers and HER2 related tumors with basal-like characteristics and further suggest that there is an apparent strong lineage-specific component to this phenotype.

## Discussion

Breast tumors are currently characterized in the clinic based on tumor size, visual characteristics, and a limit number of histochemical markers including estrogen receptor, progesterone receptor, and HER2 receptor status. Although these measures provide information about tumor properties, they offer minimal insight into the underlying biological mechanisms and provide only limited guidance in the development of therapeutic strategies. This can be best illustrated by the differential response of patients to treatment with the HER2 inhibitor Herceptin; while HER2 negative patients do not response to Herceptin, only fraction of HER2+ patients respond to this treatment suggesting that additional unrecognized heterogeneity must exist within these patients and that more sophisticated strategies must be employed to investigate tumor heterogeneity and to develop therapeutic regimens.

Consistent with this hypothesis, a number of recent studies have illustrated the heterogeneity of tumors driven by HER2 amplification. These studies have demonstrated variability in patterns of copy number variation, DNA methylation, and global gene expression [11,12]. Moreover, molecular subtypes based on the analysis of gene expression patterns [13] as well as more complex analyses of patterns of oncogenic and tumor suppressor pathway activities [14] have identified classes that can subdivide HER2 related tumors and provide further insight into the molecular and clinical heterogeneity of HER2 related tumors beyond the ERBB2+ molecular classification. As previously discussed, we recently reported a quantitative tumor classification scheme based on patterns of pathway-associated signatures that can better match the complexity of the tumor, and in doing so, identified two subgroups characterized by high levels of HER2 pathway activity [14]. In the current study, we have utilized this framework to analyze non-genetic factors in the context of genetic factors to define the relationship between these two distinct sources of tumor heterogeneity. We found a high degree of specific co-regulation between non-genetic stresses and oncogenic signaling pathways. Many of the co-regulated and correlated pathways observed in the tumors are consistent with previous findings to validate our approach. For example, the strong co-regulation of hypoxia/EGFR/TNFα/TGFβ/STAT3 pathways clearly distinguishes between two groups of HER2 related tumors with significant differences in the pathway activities of these pathways. By analyzing patterns of non-genetic stress factors, we demonstrate significant variation in patterns of predicted tumor microenvironment activity in two HER2 related subgroups classified by patterns of oncogenic pathway activity. The HER2 related tumors that are characterized by high levels of predicted hypoxia response display enhanced levels of hypoxia-related genes including *HIF-1α*, exhibit similar patterns of pathway activity and gene expression as compared to basal-like breast tumors. In contrast, the HER2 related tumors that express low levels of hypoxia have oncogenic features in common with luminal-like tumors. As illustrated in Figure 5, the correlation between hypoxia-related gene expression, HIF gene expression, and basal-like features is common across HER2 related tumors irrespective of the classification strategy used to identify these tumors; tumors classified by patterns of oncogenic pathway activity (subgroups 7/10), by the intrinsic gene list (ERBB2+) or by IHC (HER2+) all demonstrate this characteristic. Moreover, we demonstrate that breast tumors in general demonstrate this pattern of coincidental gene expression where the increased expression of basal-like genes correlates with the expression of HIF and hypoxia-related genes. These results are supported by, and are consistent with, several studies that suggest that the observed heterogeneity in ER status among HER2 related tumors is consistent with differences in the basal and luminal origins of HER2-driven breast cancers [3,11,63] as well as additional studies which report that basal-like tumors have higher levels of the hypoxia gene expression program compared to luminal breast tumors [55,64]. Finally we demonstrate that *HIF-1α *expression levels are higher in basal-like tumors, HER2 related tumors with high hypoxia, and normal basal breast epithelial cells and that *HIF-1α *regulates the expression of hypoxia-induced genes. Our data support the idea that the differences in the hypoxic response and mRNA level of hypoxia regulators may be an intrinsic cell lineage property associated with the origin of the tumor progenitor cell. Consistent with this idea, it was recently suggested that HER2 related tumors arise from late luminal progenitor cells, an intermediate differentiation stage between luminal progenitor cells from which basal-like tumors arise and differentiated luminal cells from which luminal A and B tumors arise [66]. Our results, in conjunction with others [13] suggest that some HER2 related tumors may arise from early luminal progenitor cells that are more basal-like in nature whereas other HER2 related tumors may arise from more differentiated, late luminal progenitor cells resulting in tumors with more luminal-like characteristics.

The positive correlation between both *HIF-1α *and *EPAS1 (HIF-2α) *mRNA levels and overall breast tumor hypoxia response suggests a role for transcriptional regulation of these hypoxia regulators. Our findings which demonstrate that elevated expression of *HIF-1α *mRNA in the basal and HER2 related breast tumors with basal-like features, including subgroup 7 tumors, correlates with hypoxia response are consistent with recent studies reporting higher mRNA levels of *HIF-1α *and *EPAS1 (HIF-2α) *in glioma stem cells and renal epithelial cells [24,25]. The consistent pattern of higher mRNA levels of hypoxia-inducible factors in the less differentiated glioma stem cells [25], hematopoietic stem cells [67,68] and basal mammary epithelial cells (this study) suggests that the differentiation status of these cells can be coupled with the fine-tuning of the hypoxia response and that these changes may be responsible for their distinct sensitivity to oxygen and metabolic requirements. It has also been reported that pO_2 _and hypoxia-inducible factors play important roles in the differentiation processes of several different cell types [69,70] and has been shown to induce de-differentiation of breast cancer cells promoting invasion [71].

Our findings also have translation potential and important therapeutic implications. Gaining a more complete understanding of the biological mechanisms regulating oncogenesis will enable the development of more rational therapeutic strategies to enhance therapeutic efficacy. The cellular origin of HER2 related tumors, the patterns of oncogenic pathway activity, and the contributions of microenvironmental stresses may be clinically relevant since differences in levels of PI3K, EGFR, hypoxia, or MET activities in tumors have been reported to contribute to various therapeutic responses to HER2-targeting and other therapies, such as Trastuzumab (Herceptin). The influence of these factors on response can be direct as in the case of PI3K [59,72], or indirect, as in the case of development of drug resistance under hypoxia [18]. Aberrant MET expression has also been shown to confer resistance to the EGFR inhibitor, Gefitinib [73]. Therefore, co-activation of these pathways in subgroup 7 tumors provides further justification for the development and investigation of therapeutic regimens that incorporate compounds targeting more than one oncogenic pathway in order to increase efficacy and reduce resistance.

## Conclusions

We have incorporated non-genetic (microenvironmental stresses) with genetic (oncogenic and tumor suppressor genes) factors together in a pathway-based classification framework to further dissect and identify biological mechanisms underlying tumor heterogeneity. We validate this approach by investigating the basis for the distinct patterns of oncogenic pathway activity and microenvironmental conditions of two HER2 related tumor subgroups and found significant similarities with many features distinguishing between the basal and luminal intrinsic subtypes of breast cancers. By examining tumors identified as HER2 related by patterns of oncogenic pathway activity (subgroups7/10), tumors defined as HER2 related by the intrinsic gene list (ERBB2+), or tumors that are HER2+ by IHC, we demonstrate that these features are common to HER2 related tumors irrespective of the strategy used to characterize them as HER2+. By revealing the role of *HIF-1α *in regulating the hypoxia response and showing that this mechanism, as well as the expression of additional features, link HER2 related tumors in subgroup 7 with basal-like breast tumors and subgroup 10 tumors with luminal like breast tumors, respectively, we identify additional oncogenic mechanisms and HER2 related tumor heterogeneity that was not previously evident. While our current studies focus only on a subset of chemical stresses, a similar approach is likely to be applicable for incorporating the mechanical factors, physical forces, interstitial pressure, paracrine milieu and other non-genetic conditions that may impact tumor phenotypes. Such strategies are scalable to incorporate additional sources of information to investigate and discover cross-talk between different pathways.

## Abbreviations

ARNT: aryl hydrocarbonreceptor nuclear translocator; BFRM: Baysian Factor Regression Modeling; CA9: carbonic anhydrase IX; cMET: met proto-oncogene; DEC1: deleted in esophageal cancer; EGFL3: egl nin homolog 3; EGFR: epidermal growth factor receptor; EP300: e1A binding protein p300; EPAS1: endothelial PAS domain containing protein 1; ER: estrogen receptor; ERBB2: v-erb-B2 erythroblastic leukemia viral oncogene homolog 2; GATA3: GATA binding protein 3; Glu(-): Glucose depletion; Glut1: glucose transporter type 1; HER2: Human epidermal growth factor receptor 2; HIF-1α: hypoxia-inducible factor 1 alpha; HIF-2α: hypoxia-inducible factor 2 alpha; IFN-α: interferon alpha; IGF1R: insulin growth factor 1 receptor; INF-γ: interferon gamma; Jun: Jun proto oncogene; KRT5: 17, 18, 19, cytokeratin 5, 17, 18, 19; LDHA: lactatedehydrogenase A; Pl3k: phosphatidylinositol 3-kinase; PR: progesterone receptor; STAT3: signal transducer and activator of transduction 3; TGF-β: tumor growth factor beta; TNF-α: tumor necrosis factor alpha; TXNIP: thioredoxin interacting protein; VEGFA: vascular endothelial growth factor A.

## Competing interests

The authors declare that they have no competing interests.

## Authors' contributions

MLG, HNK, JRM and JTC conceived and designed the study. MLG and HNK generated and analyzed data. KLB, MWD, and JRM provided unpublished reagents and data. MLG, HNK and JTC wrote the manuscript. All authors read and approved the final manuscript.

## Supplementary Material

Additional File 1 **Supplementary methods**. This document provides additional information about the methods used in the manuscript. Click here for file

Additional File 2 **Supplemental Table S1. Summary of microenvironment signatures**. This document provides a summary of the microenvironment signatures and parameters. Click here for file

Additional File 3 **Supplemental Table S2. Summary of tumor samples**. This document provides a summary of the tumor samples used in the study. Click here for file

Additional File 4 **Supplementary Figures S1 to S18**. This document contains Supplementary Figures S1 to S18. Click here for file
