# Supplementary material for: Analysis of tumor environmental response and oncogenic pathway activation identifies distinct basal and luminal features in HER2-related breast tumor subtypes
Source: Breast Cancer Res. 2011 Jun 7;13(3):R62. doi: 10.1186/bcr2899 (PMC3218951; doi:10.1186/bcr2899)
Supplement: Additional File 1 — Supplementary methods. This document provides additional information about the methods used in the manuscript. [file bcr2899-S1.PDF]

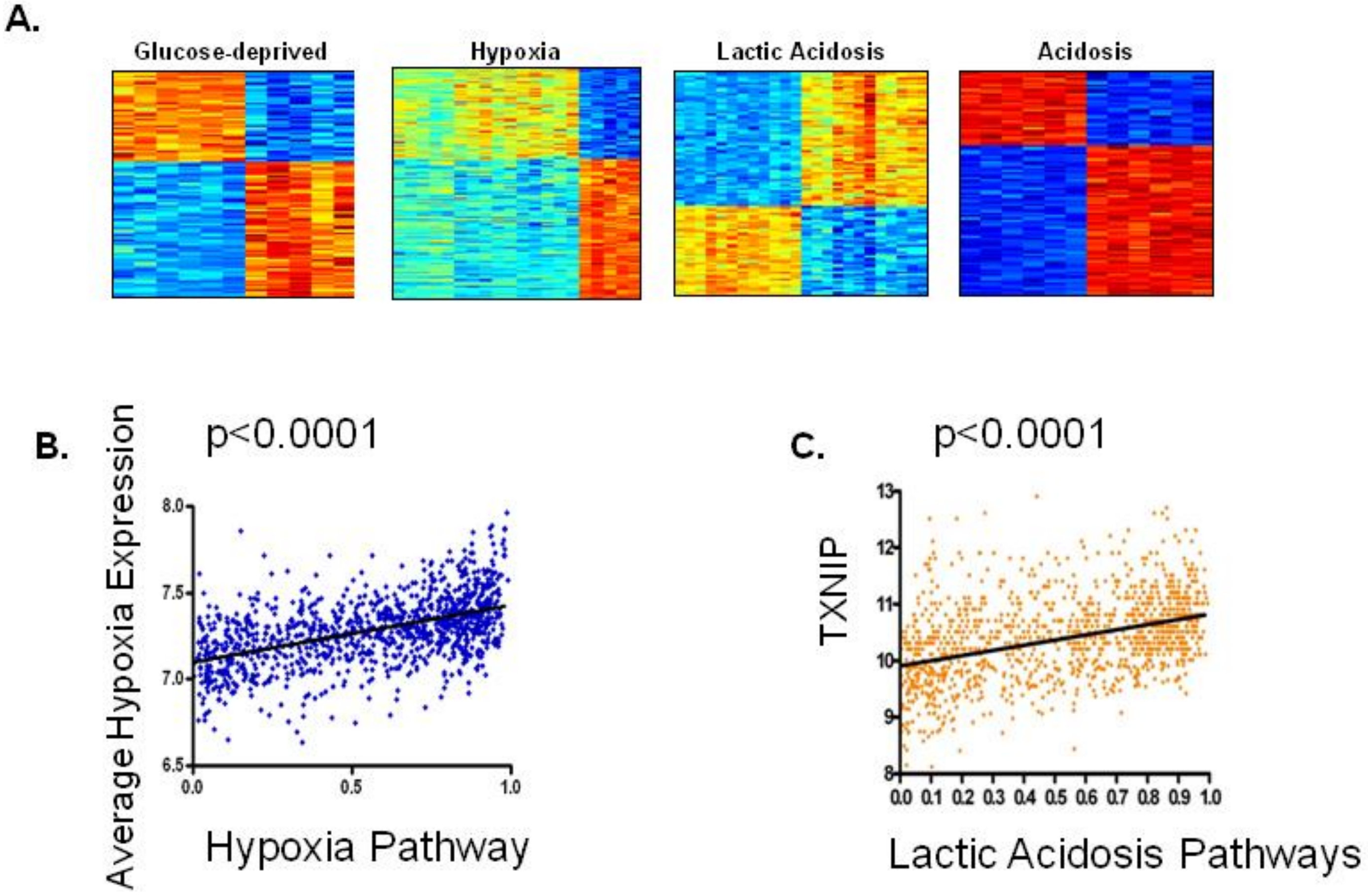

Supplementary Figure S1: The heatmaps (A) of the gene signatures of the indicated microenvironmental stresses. (B) The predicated hypoxia pathways are validated by the strong association with average expression of previously defined hypoxia-inducible genes [44] in the dataset of 1,143 breast tumors. (C) The predicted lactic acidosis pathways are validated by strong association with the expression *TXNIP*, as previously reported [24], in the dataset of 1,143 breast tumors.

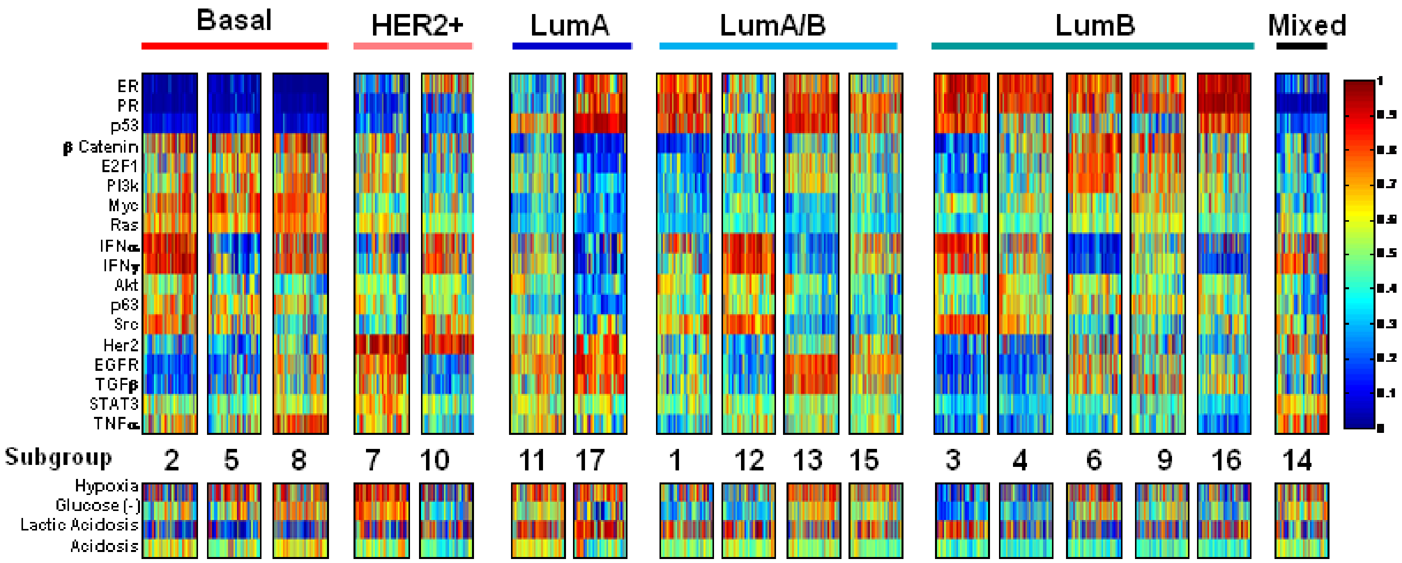

Supplementary Figure S2: The heatmap of predicted microenvironment stress activities shown in the context of 17 breast cancer subgroups defined by oncogenic pathways [14]. Red indicated a high probability of pathway activity; blue, a low probability of pathway activity.

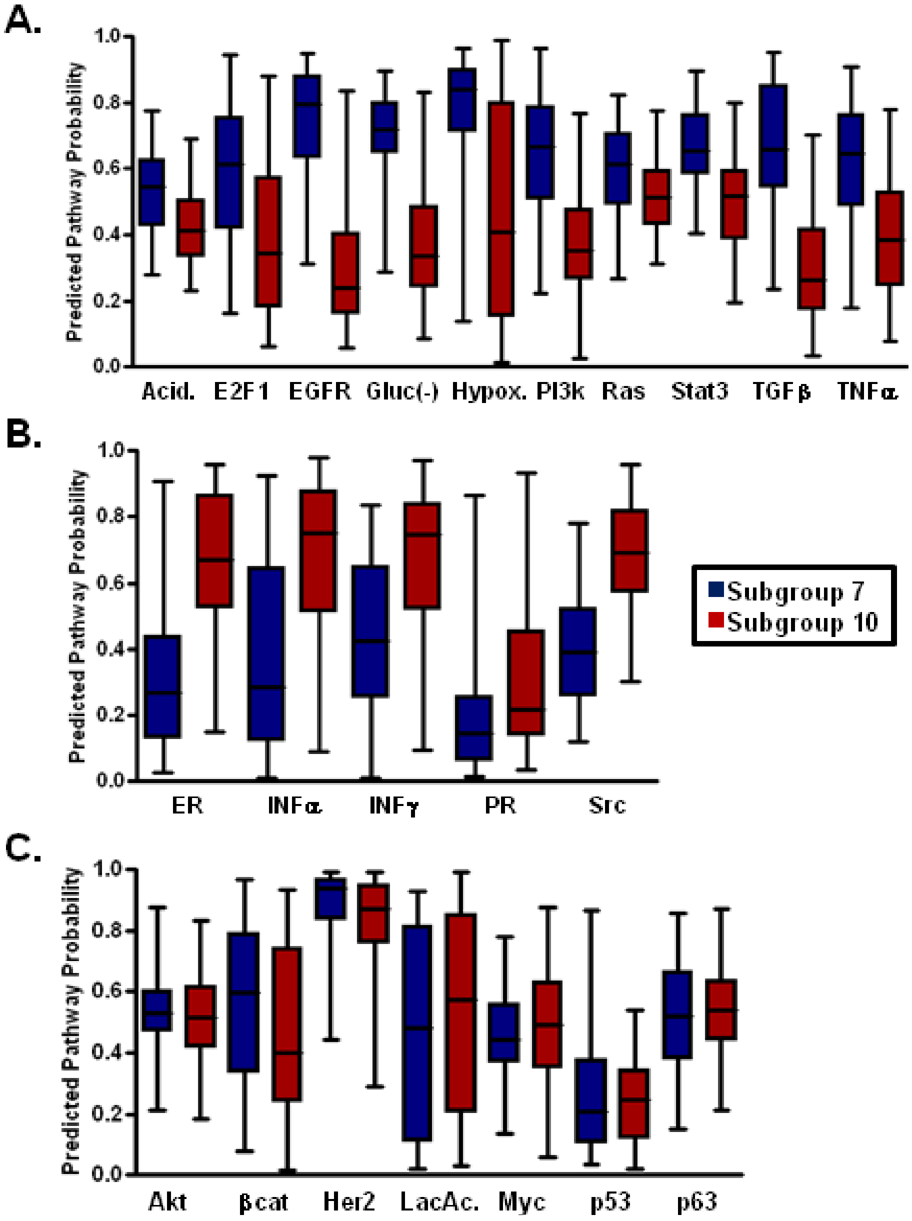

Supplementary Figure S3: (A) Box and whisker plot identifying pathways significantly up-regulated in subgroup 7: acidosis (p<0.0001, unpaired t-test), E2F1 (p<0.0001), EGFR (p<0.0001), glucose depletion (p<0.0001), hypoxia (p<0.0001), PI3k (p<0.0001), Ras (p=0.0013), STAT3 (p<0.0001), TGFβ (p<0.0001), TNFα (p<0.0001). (B) The pathways significantly up-regulated in subgroup 10: ER (p<0.0001, unpaired t-test), IFNα (p<0.0001), IFNγ (p<0.0001), PR (p=0.0015), and Src (p<0.0001). (C) The pathways which are not significantly different among the two HER2 related subtype: βcatenin, Her2, Lactic Acidosis, c-myc, p53 and p63.

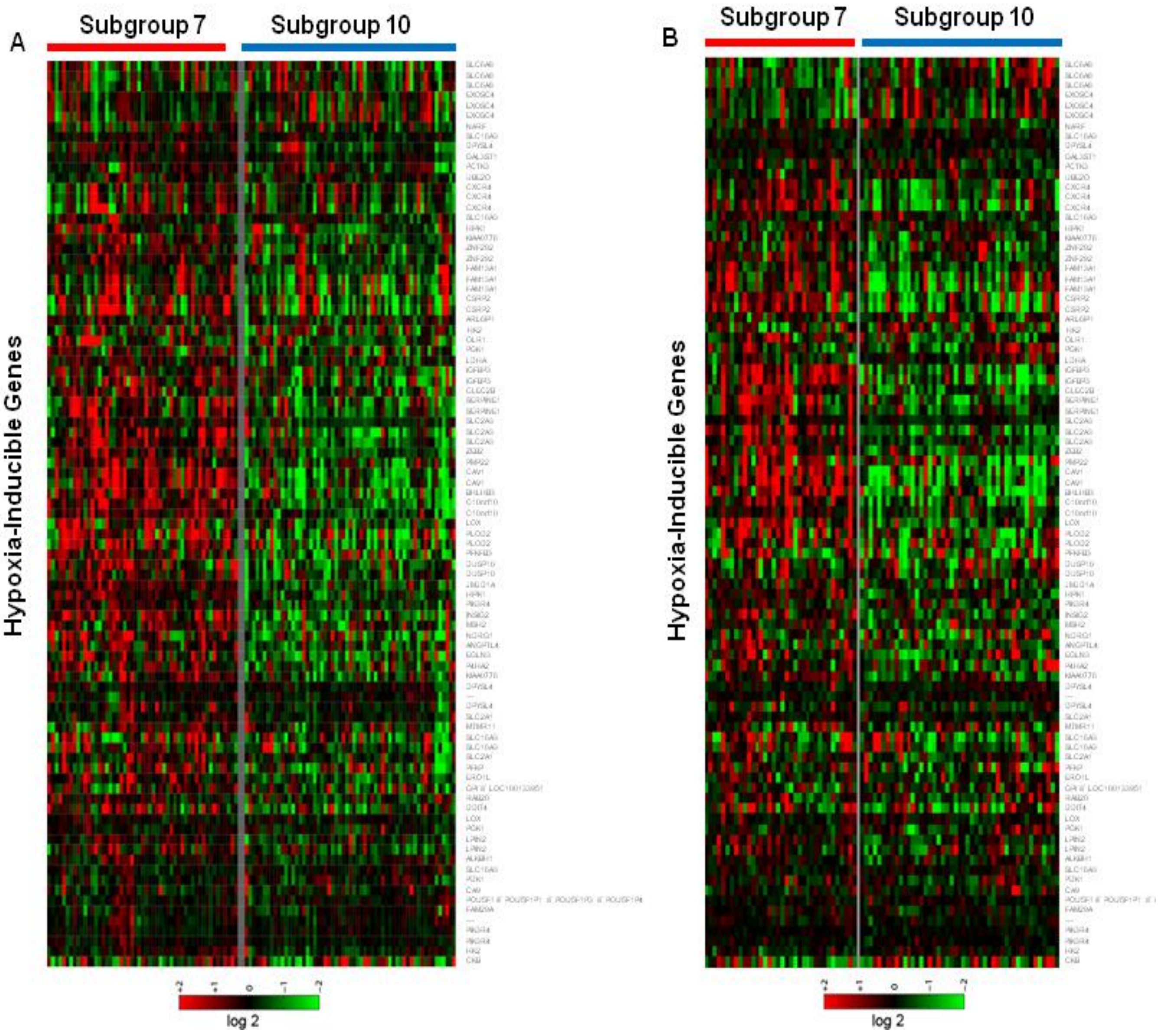

Supplemental Figure S4: High resolution heatmap showing the expression level of hypoxia-induced genes in subgroups 7 and 10 tumors in (A) the primary dataset and (B) the validation dataset. Specific genes analyzed are reported and the associated Affymetrix U133 probes are reported (in the same order) in the Supplemental Methods.

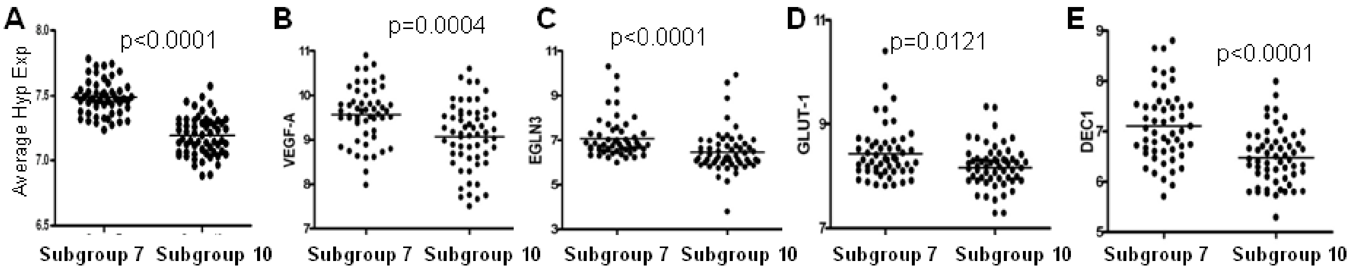

Supplementary Figure S5: HER2 related subgroup 7 tumors show higher expression levels of hypoxia-inducible genes compared to subgroup 10 tumors. (A) The average expression levels of hypoxia-inducible genes were significantly higher in the HER2-basal subtype, as further shown by a significantly higher mRNA levels of hypoxia-induced genes (B) *VEGFA* (p=0.0004), (C) *EGLN3* (p<0.0001), (D) *GLUT-1* (p=0.0121), and (E) *DEC1* (p<0.0001), all unpaired t-test.

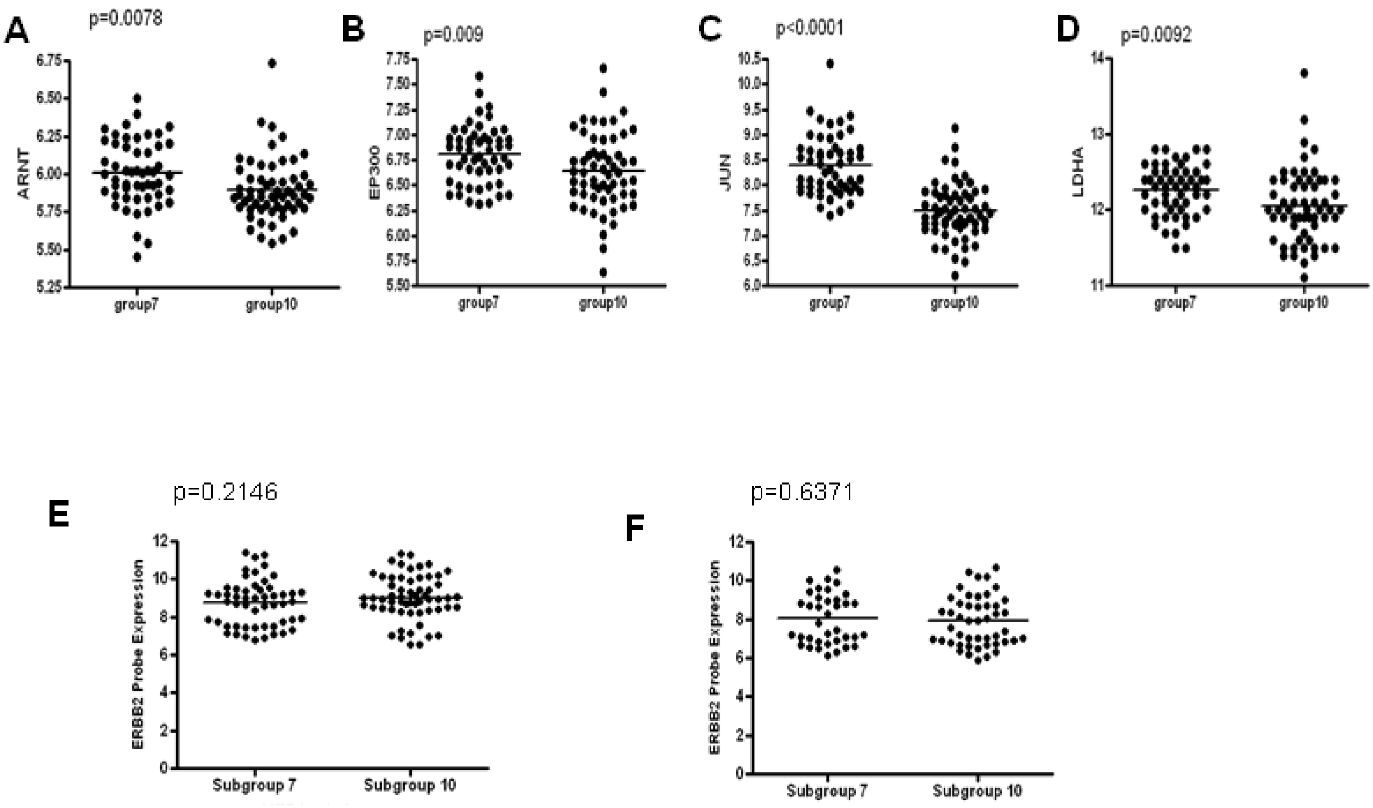

Supplementary Figure S6: Subgroup 7 tumors demonstrate significantly higher levels of other regulators of hypoxia responses, including (A) *ARNT* (p=0.0078), (B) *EP300* (p=0.009), (C) *JUN* (p<0.0001), (D) *LDHA* (p=0.0092). The expression values of *ERBB2* mRNA were not significantly different among the two HER2 related tumor subtypes in the primary (E, p=0.2146) and validation (F, p=0.6371) datasets.

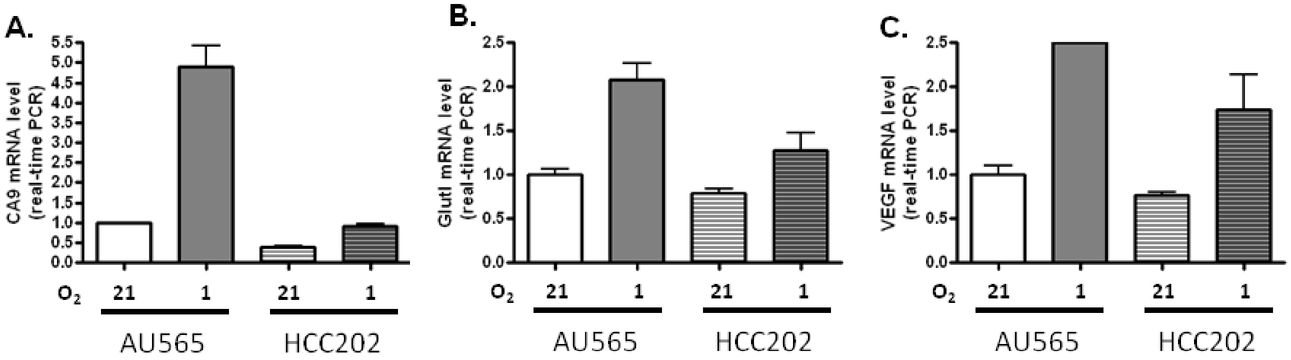

Supplementary Figure S7: Subgroup 7 cell line AU565 shows higher expression levels of the hypoxia-inducible genes (A) *CA9*, (B) *GLUT-1* and (C) *VEGF-A* when compared with HCC202 cell line (subgroup 10).

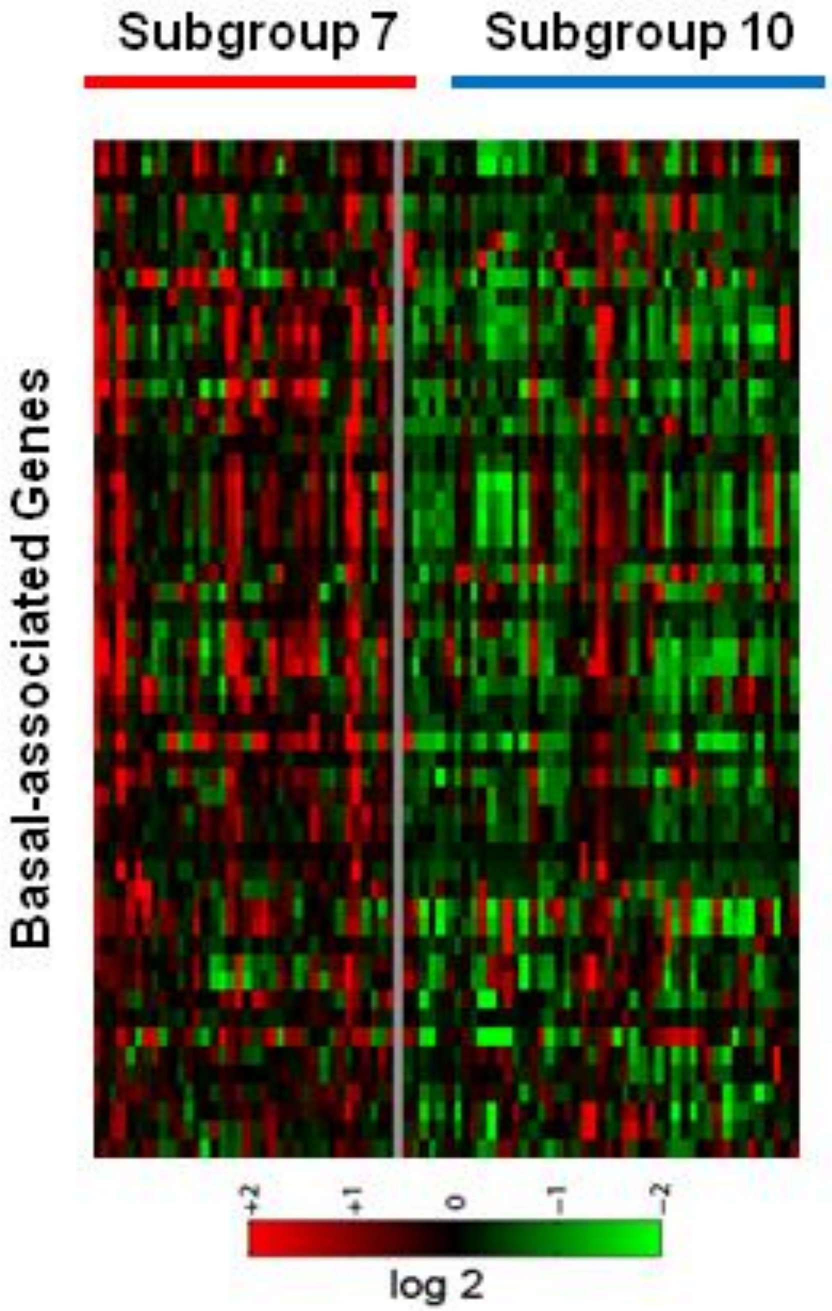

Supplementary Figure S8: High resolution heatmap showing the expression level of basal specific genes in subgroups 7 and 10 tumors in the validation dataset. Specific genes analyzed and the associated Affymetrix U133 probes are reported (in the same order) in the Supplemental Methods.

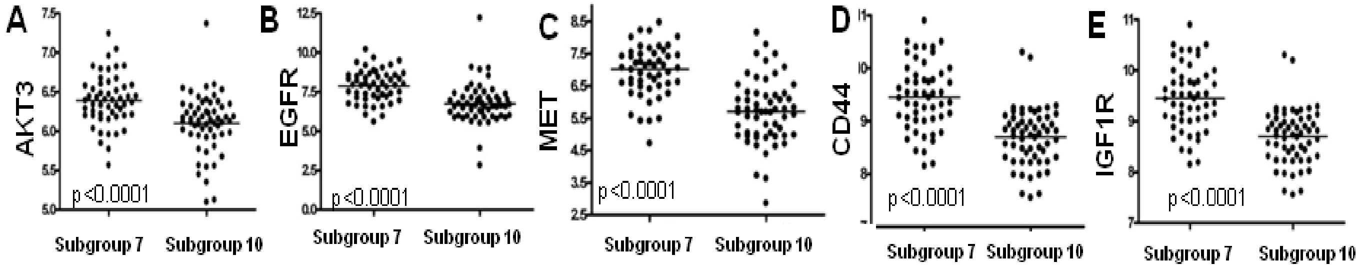

Supplementary Figure S9: HER2 related tumors in subgroup 7 show higher levels ($p<0.0001$) of (A) *AKT3*, (B) *EGFR*, (C) *MET*, (D) *CD44* and (E) *IGF1R* expression as compared to HER2 related tumors in subgroup 10.

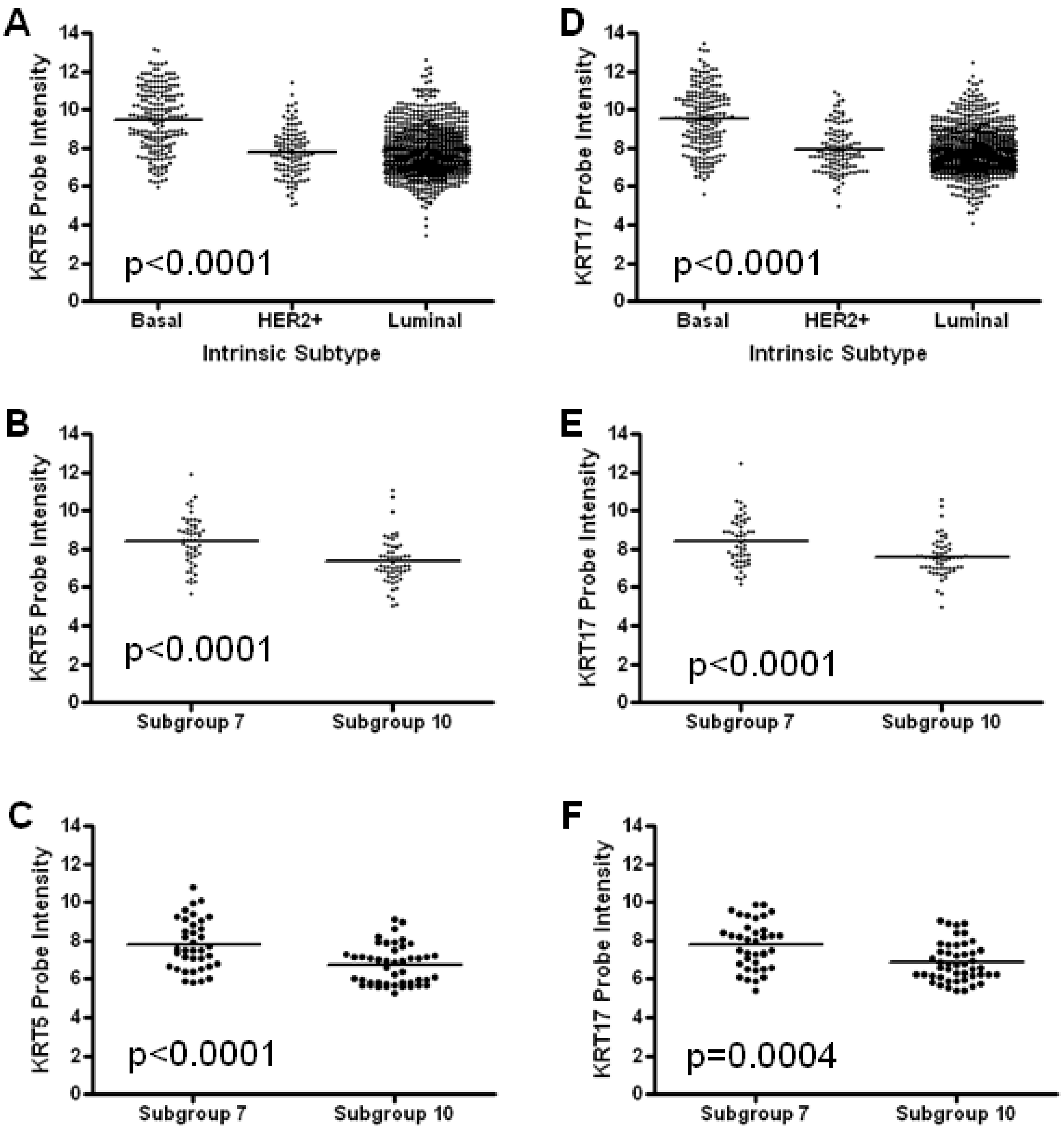

Supplementary Figure S10: (A) The expression level of basal cytokeratin marker *KRT5* is higher in basal tumors than luminal breast tumors from the primary dataset of 1,143 samples ($p<0.0001$). (B-C) HER2 related subgroup 7 tumors show higher levels of basal cytokeratin markers *KRT5* in the (B) primary dataset ($p<0.0001$) and (C) validation dataset ($p<0.0001$). (D) The expression levels of basal cytokeratin marker *KRT17* is higher in basal tumors than luminal breast tumors from the validation dataset of 547 tumors ($p<0.0001$). (E, F) HER2 related subgroup 7 tumors show higher levels of basal cytokeratin markers *KRT17* in the primary (E, $p<0.0001$) and validation dataset (F, $p=0.0004$).

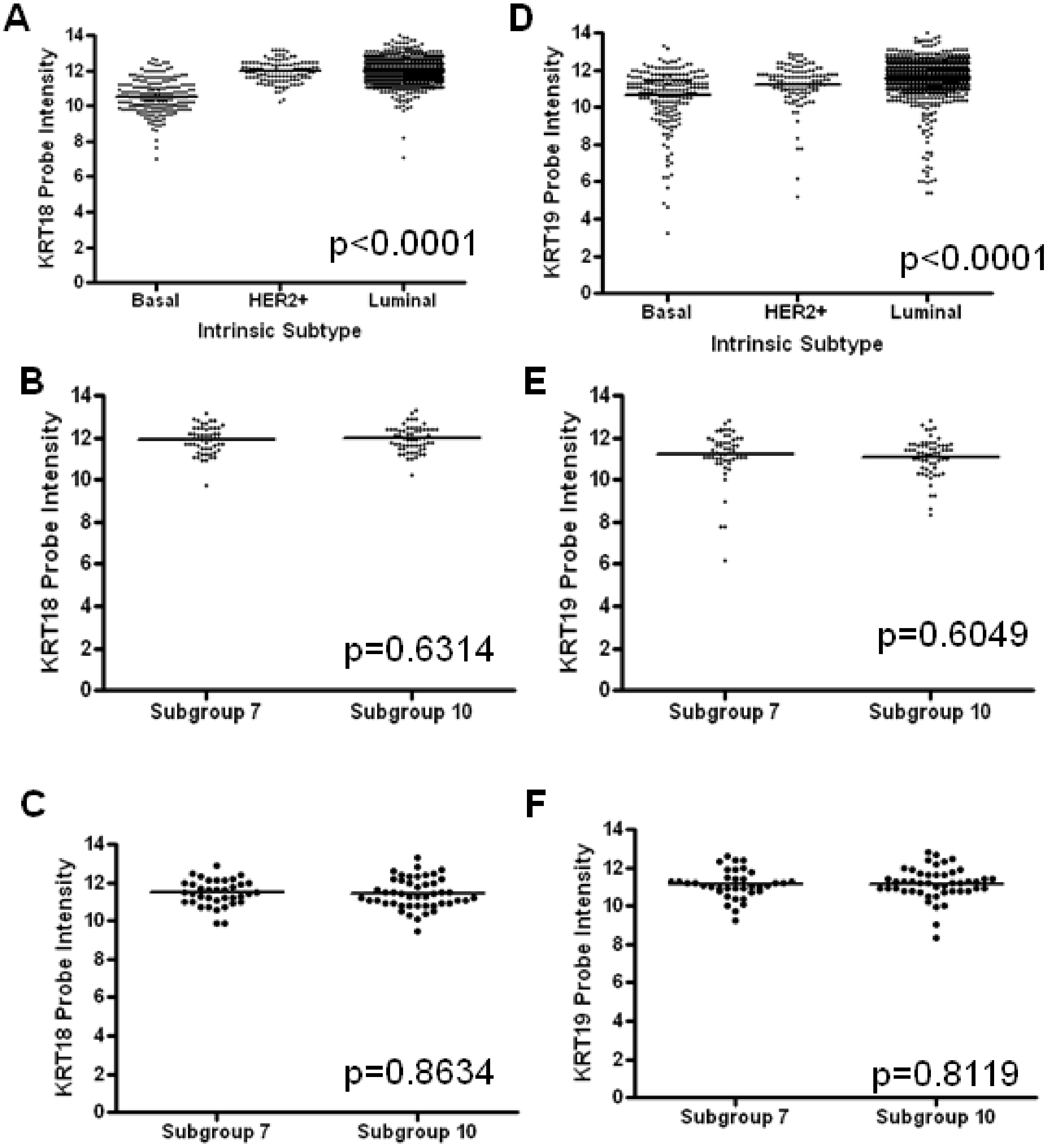

Supplementary Figure S11: (A) The expression level of luminal cytokeratin markers KRT18 is greater in luminal breast tumors as compared to basal breast tumors in the primary dataset of 1,143 samples (p<0.0001). (B, C) No significant difference in *KRT18* levels exist between HER2 related subgroups 7 and 10 in the primary (B, p=0.6314) and validation dataset (C, p=0.8634). (D) The expression level of luminal cytokeratin markers *KRT19* is greater in luminal breast tumors as compared to basal breast tumors in the primary dataset (p<0.0001). (E, F) No significant difference in *KRT19* levels exist between HER2 related subgroups 7 and 10 in the primary (E, p=0.6049) and validation dataset (F, p=0.8119).

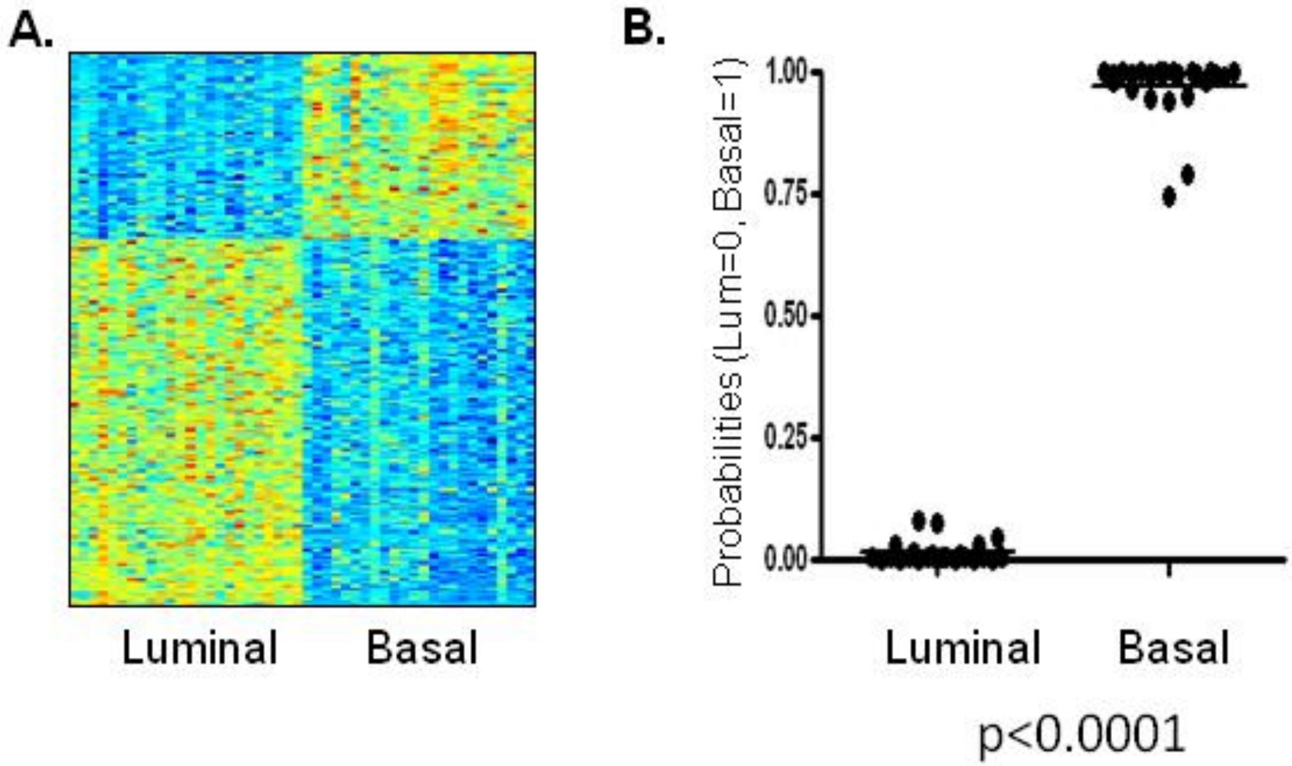

Supplementary Figure S12: (A) Heatmap depicting the gene expression signature identifying differences between the luminal and basal breast cancer cell lines. (B) Leave-one-out cross validation of the luminal-basal training data in the basal and luminal cancer cell lines.

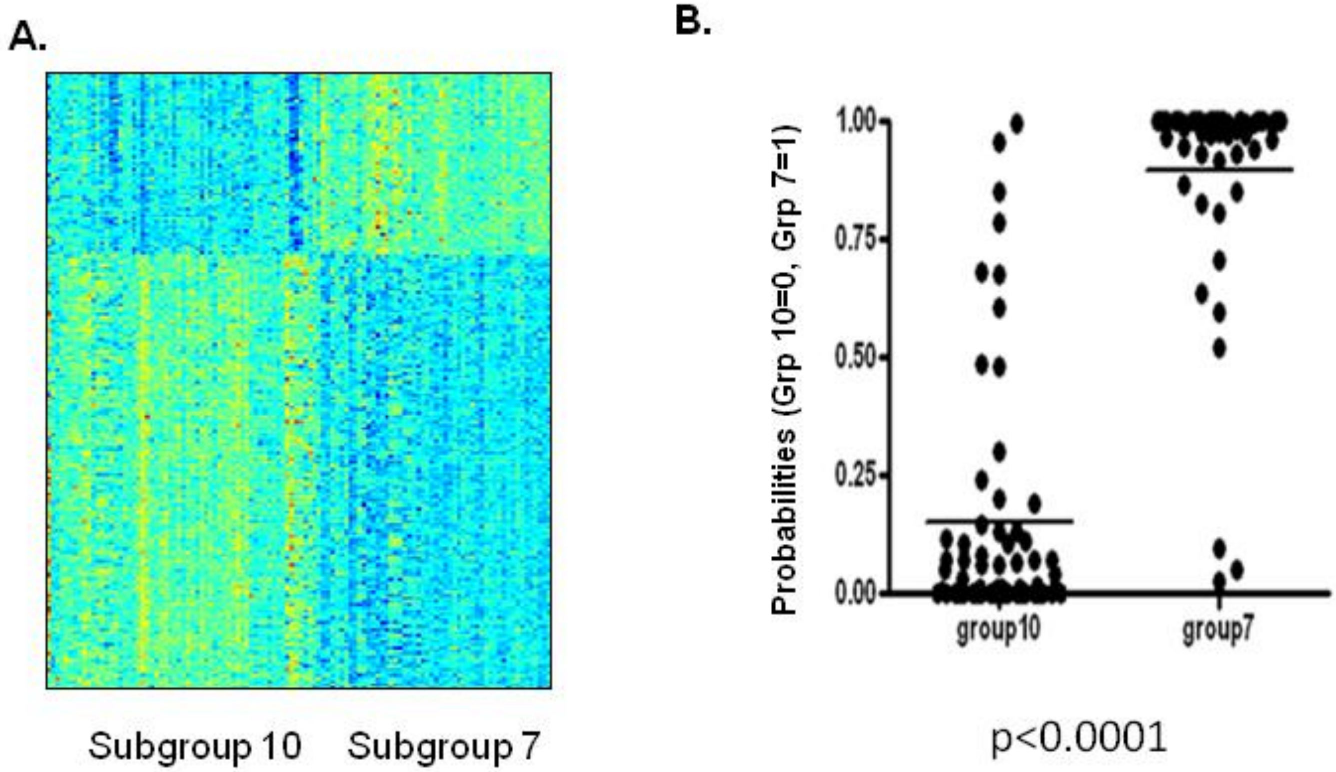

Supplementary Figure S13: (A) Heatmap depicting the gene expression signature identifying differences between subgroups 10 and 7 of HER2 related tumors. (B) Leave-one-out cross validation of the training models in the subgroups 10 and 7 of HER2 related tumors.

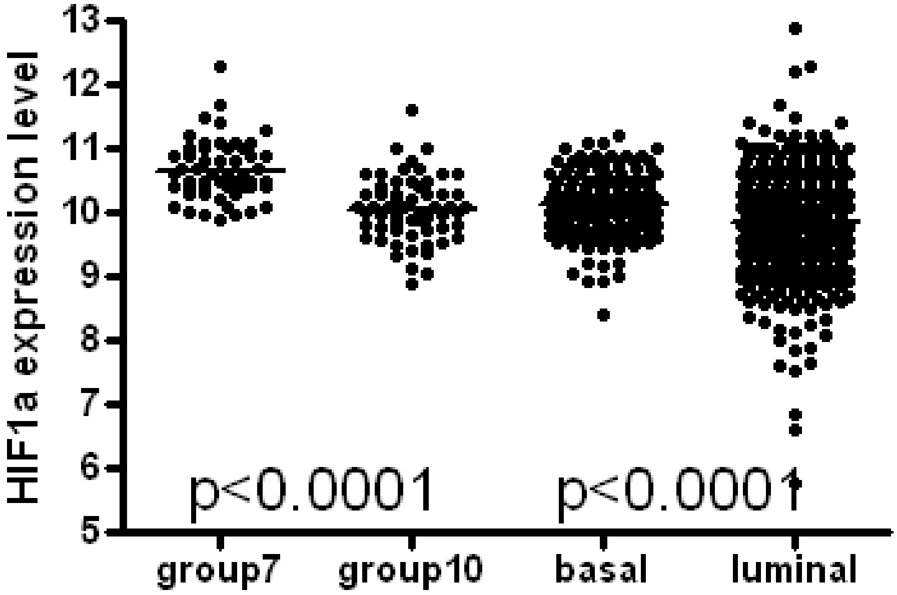

Supplementary Figure S14: Subgroup 7 (p<0.0001) and basal (p<0.0001) tumors show significantly higher levels of *HIF1α* gene expression than subgroup 10 or luminal tumors, respectively.

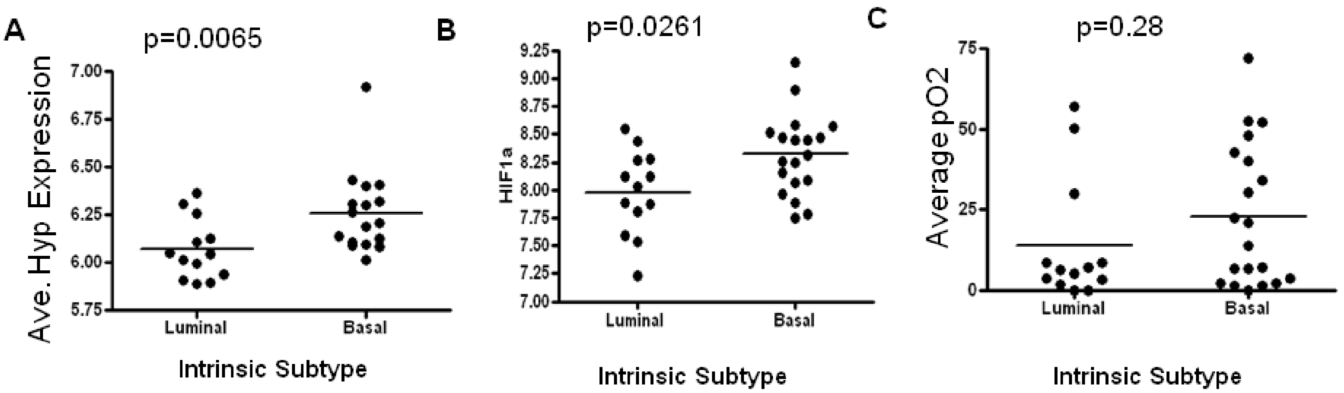

Supplementary Figure S15: The higher level of hypoxia response and *HIF-1α* mRNA levels in the basal-like tumors. In an expression dataset with both gene expression and intra-tumor pO2 [33], the average expression of hypoxia-inducible genes (A), and the expression levels of *HIF-1α* mRNA (B) and measured pO2 (C) in the basal and luminal-type breast tumors. All the expression levels were shown in log_2 scale.

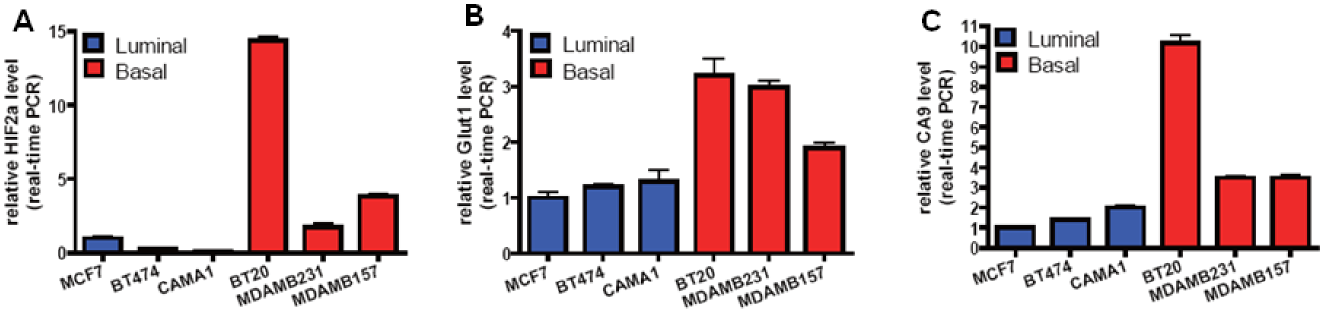

Supplementary Figure S16: The expression levels of HIF-2α mRNA (A) and two hypoxia-inducible genes (B-*VEGFA* and C-*CA9*) in the indicated luminal-like (blue) and basal-like (red) breast cancer cell lines.

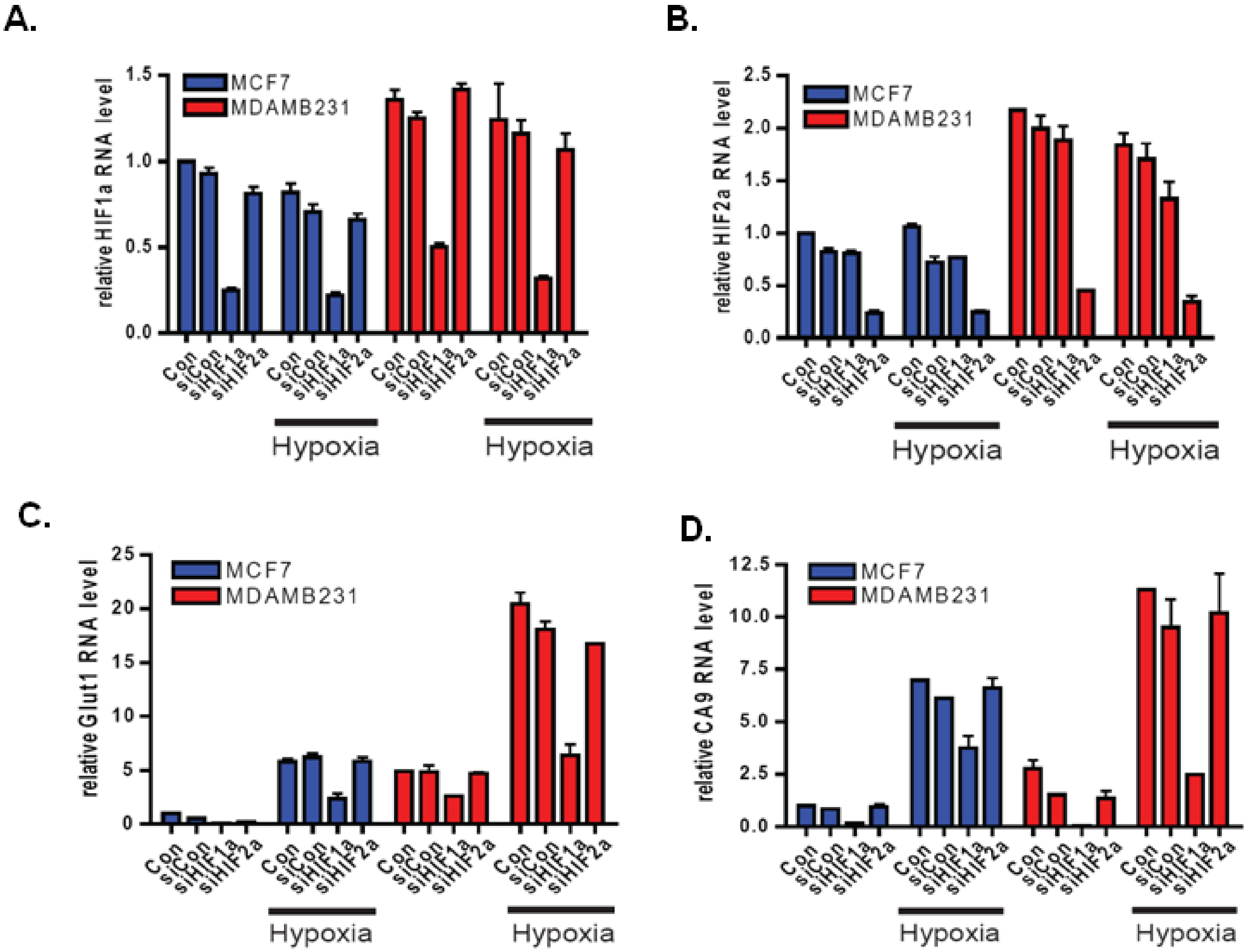

Supplementary Figure S17: The expression levels of (A) *HIF-1α* and (B) *EPAS1 (HIF-2α)* in MCF-7 (luminal-like) and MDA-MB231 (basal-like) cell lines after treatment with control siRNA or siRNA against HIF-1α , or EPAS1 (HIF-2α) under normoxia and hypoxia . (C) *Glut1* and (D) *CA9* expression levels in MCF7 and MDA-MB231 cells following treatment with control siRNA or siRNA against HIF-1α or EPAS1 (HIF-2α) under normal and hypoxic growth conditions.

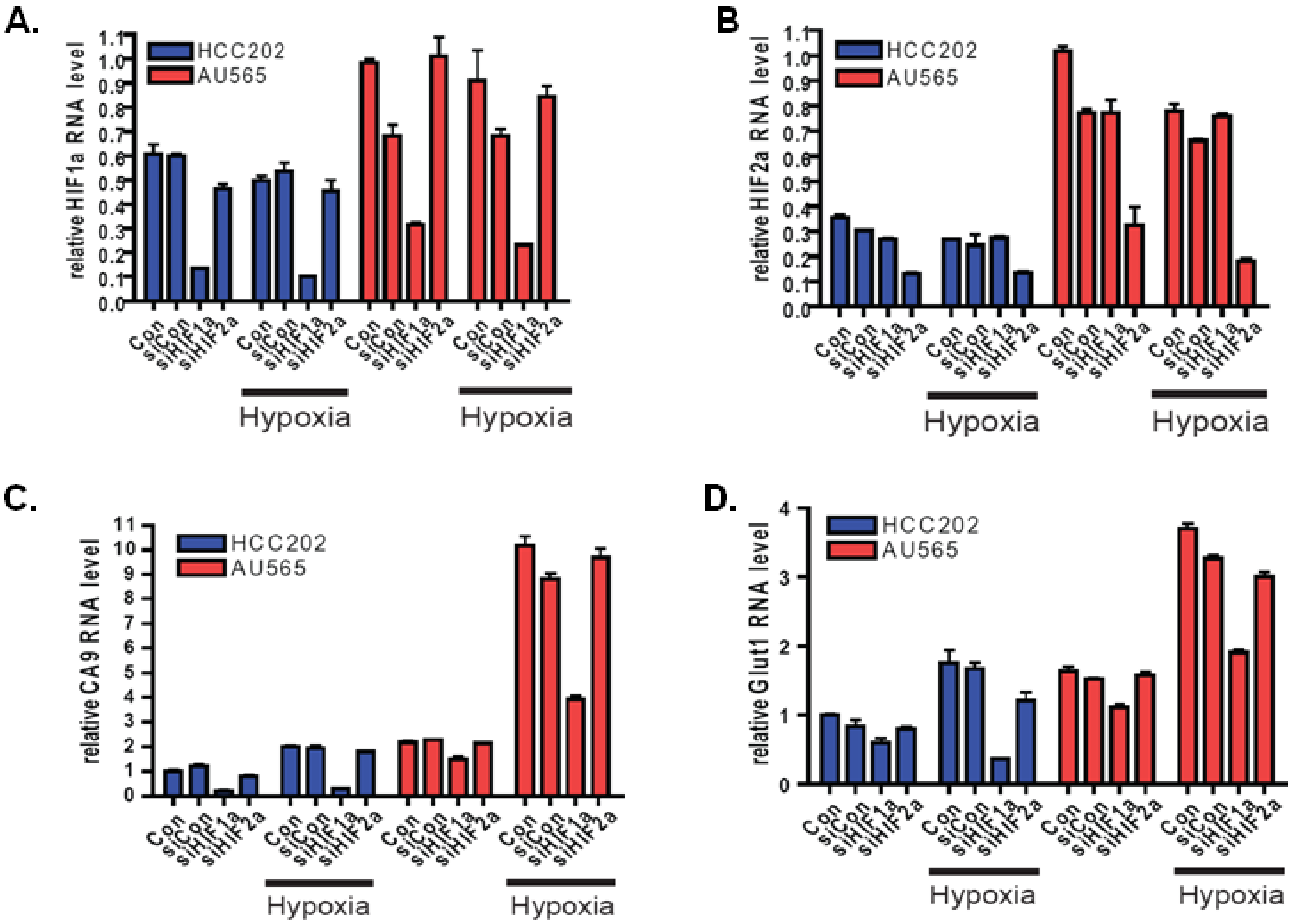

Supplemental Figure S18: The expression levels of (A) *HIF-1α,* (B) *EPAS1 (HIF-2α)*, (C) *CA9* and (D) *Glut1* in the AU565 (subgroup 7) and HCC202 (subgroup 10) after treatments with the indicated control siRNA or siRNA against HIF-1α or HIF-2α under normoxia and hypoxia.
