# Supplementary material for: Analysis of tumor environmental response and oncogenic pathway activation identifies distinct basal and luminal features in HER2-related breast tumor subtypes
Source: Breast Cancer Res. 2011 Jun 7;13(3):R62. doi: 10.1186/bcr2899 (PMC3218951; doi:10.1186/bcr2899)
Supplement: Additional File 2 — Supplemental Table S1. Summary of microenvironment signatures. This document provides a summary of the microenvironment signatures and parameters. [file bcr2899-S2.PDF]

SUPPLEMENTAL METHODS

Code Narrative for: *Analysis of tumor environmental response and oncogenic pathway activation identifies distinct basal and luminal features in HER-related breast tumor subtypes.*

This document is a step-by-step narrative of the MATLAB based methods and strategies performed in "Integrative pathway analysis identifies HER2+ tumor subtypes with basal and luminal cell features". These analyses were performed using MATLAB release 2009a with the Bioinformatics and Statistical toolboxes installed.

Analyses were performed using a Dell desktop computer with a 2.4GHz Intel DuoCore processor, 2Gb RAM, and running Microsoft Windows XP Professional 2002.

All gene expression data used in the study were arrayed on the Affymetrix HG-U133 platform.

The normalized training data used in this study are publicly available at:
http://data.genome.duke.edu/Gatza_Kung

I. Development and Validation of Pathway Signatures.

This section will detail the steps taken to develop each of the gene expression signatures described in the study. We will specifically detail the sources of data, the methods used to prepare the data, and the process used to generate the initial pathway signature. Many of these steps will be redundant between signatures.

ONCOGENE and TUMOR SUPPRESSOR SIGNATURES

The methods used to generate the 18 oncogene and tumor suppressor pathway signatures have been described in great detail by Gatza *et al.* [14] and the training data and parameters are publicly available at http://data.genome.duke.edu/breast_subgroup.

Binreg ver2.0 is available at: http://www.duke.edu/~binbarry/BINREG/.

ACIDOSIS

A. Data sources:

Human mammary epithelial cells (HMEC) were cultured in MEGM (Cambrex) and growth factors were withdrawn 24 hours prior to exposure to stress conditions. Six replicates of HMECs were exposed to normal conditions while an additional six replicates were exposed to media with a pH of 6.7 (HCl) (GSE9649). These data have been previously reported in Chen *et al.* [44].

B. Data Preparation:

- RNA was collected from the described experimental conditions and hybridized to Affymetrix U133+2 arrays.
- The expression data was downloaded from GSE9649
- The downloaded .CEL files were RMA normalized using Affymetrix Expression Console (ver1.0), filtered to contain only the U133A probe sets, and the resulting output saved as acidosis_train_rma.txt.
- In order to enable this file to be utilized by BINREG (ver2), acidosis_train_rma.txt was opened in Microsoft Excel and the sample labels were changed from normal to 0 and acidosis to 1. This file was saved as acidosis_rma.xlsx.

C. Generation of ACIDOSIS Signature:

- In MATLAB, the BINREG (ver2.0) GUI window was opened using the command >>binreg.
- In the GUI window, acidosis_rma.xlsx was imported.
- The parameters were set as summarized in Supplemental Table 1.
- BINREG was then run to generate the signature. The statistical methodology used in this analysis is described in Part II of the Supplemental Methods section entitled Analysis of Expression Data for Predicting Pathway Activity.
- The ACIDOSIS signature probe list and regression weights are reported by BINREG in the output file entitled genecoefficient.txt and the image intensity display (or heatmap) of these values is reported in output figure 1. The ACIDOSIS signature gene list is reported in Supplemental Table 1 of the manuscript and the ACIDOSIS signature heatmap is depicted in Supplemental Figure 1 of the manuscript.
- The formal leave-one-out cross validation used to examine the stability and predictive capability of the model is reported by BINREG in the file entitled crossvalidation.txt and the corresponding graph is reported in BINREG figure 6.

GLUCOSE DEPLETION

A. Data sources:

Human cancer cell lines (GSE13548) exposed to normal growth media or under glucose depleted conditions described by Saito *et al.* [48]. Samples grown under normal conditions were: GSM341398, GSM341399, GSM341404, GSM341405, GSM341406, and GSM341407. Samples grown under glucose depleted conditions were: GSM341389, GSM341401, GSM341402, GSM341413, and GSM341426.

B. Data Preparation:

- Affymetrix U133+2 .CEL files of the cancer cell lines grown under normal or glucose depleted conditions were downloaded from GSE13548.
- The .CEL files were RMA normalized using Affymetrix Expression Console (ver1.0), filtered to contain only the U133A probes sets and saved as gluc_train.txt.
- In order to enable this file to be utilized by BINREG (ver2), gluc_train_rma.txt was opened in Microsoft Excel and the sample labels were changed from normal to 0 and glucose depletion to 1. This file was saved as gluc_rma.xlsx.

C. Generation of the GLUCOSE DEPLETION Signature:

- In MATLAB, the BINREG (ver2.0) GUI window was opened using the command >>binreg.
- In the GUI window, gluc_rma.xlsx was imported.
- The parameters were set as summarized in Supplemental Table 1.
- BINREG was then run to generate the signature. The statistical methodology used in this analysis is described in part II of the Supplemental Methods section entitled Analysis of Expression Data for Predicting Pathway Activity.
- The GLUCOSE DEPLETION signature probe list and regression weights are reported by BINREG in the output file entitled genecoefficient.txt and the image intensity display (or heatmap) of these values is reported in output figure 1. The GLUCOSE DEPLETION signature gene list is reported in Supplemental Table 1 of the manuscript and the GLUCOSE DEPLETION signature heatmap is depicted in Supplemental Figure 1 of the manuscript.
- The formal leave-one-out cross validation used to examine the stability and predictive capability of the model is reported by BINREG in the file entitled crossvalidation.txt and the corresponding graph is reported in BINREG figure 6.

HYPOXIA

A. Data Source:

MCF7 cells were grown under normal (21% oxygen) conditions, exposed to lactic acidosis (25mM), or exposed to glucose depletion and compared to MCF7 cells grown under hypoxic (2% oxygen) conditions for four hours.

B. Data Preparation:

- RNA was collected from the described experimental conditions and hybridized to Affymetrix U133+2 arrays (GSE19123).
- The resulting .CEL files were RMA normalized using Affymetrix Expression Console (ver1.0), filtered to contain only the U133A probe sets, and the resulting output saved as hypoxia_train_rma.txt.
- In order to enable this file to be utilized by BINREG (ver2), acidosis_train_rma.txt was opened in Microsoft Excel and the sample labels were changed from normal to 0 and hypoxia to 1. This file was saved as hypoxia_rma.xlsx.

C. Generation of the HYPOXIA Signature:

- In MATLAB, the BINREG (ver2.0) GUI window was opened using the command >>binreg.
- In the GUI window, hypoxia_rma.xlsx was imported.
- The parameters were set as summarized in Supplemental Table 1.
- BINREG was then run to generate the signature. The statistical methodology used in this analysis is described in part II of the Supplemental Methods section entitled Analysis of Expression Data for Predicting Pathway Activity.
- The HYPOXIA signature probe list and regression weights are reported by BINREG in the output file entitled genecoefficient.txt and the image intensity display (or heatmap) of these values is reported in output figure 1. The HYPOXIA signature gene list is reported in Supplemental Table 1 of the manuscript and the HYPOXIA signature heatmap is depicted in Supplemental Figure 1 of the manuscript.
- The formal leave-one-out cross validation used to examine the stability and predictive capability of the model is reported by BINREG in the file entitled crossvalidation.txt and the corresponding graph is reported in BINREG figure 6.

LACTIC ACIDOSIS

A. Data Source:

MCF7 cells were grown under normal conditions or exposed to lactic acidosis (25mM lactic acid) for 1, 4, 12 and 24 hours. Three replicates were taken at each time point.

B. Data Preparation:

- RNA was collected from the described experimental conditions and hybridized to Affymetrix U133+2 arrays (GSE19123).
- The resulting .CEL files were RMA normalized using Affymetrix Expression Console (ver1.0), filtered to contain only the U133A probe sets, and the resulting output saved as la_train_rma.txt.
- In order to enable this file to be utilized by BINREG (ver2), acidosis_train_rma.txt was opened in Microsoft Excel and the sample labels were changed from normal to 0 and lactic acidosis to 1. This file was saved as la_rma.xlsx.

C. Generation of the HYPOXIA Signature:

- In MATLAB, the BINREG (ver2.0) GUI window was opened using the command >>binreg.

- In the GUI window, la_rma.xlsx was imported.
- The parameters were set as summarized in Supplemental Table 1.
- BINREG was then run to generate the signature. The statistical methodology used in this analysis is described in part II of the Supplemental Methods section entitled Analysis of Expression Data for Predicting Pathway Activity.
- The LACTIC ACIDOSIS signature probe list and regression weights are reported by BINREG in the output file entitled genecoefficient.txt and the image intensity display (or heatmap) of these values is reported in output figure 1. The LACTIC ACIDOSIS signature gene list is reported in Supplemental Table 1 of the manuscript and the LACTIC ACIDOSIS signature heatmap is depicted in Supplemental Figure 1 of the manuscript.
- The formal leave-one-out cross validation used to examine the stability and predictive capability of the model is reported by BINREG in the file entitled crossvalidation.txt and the corresponding graph is reported in BINREG figure 6.

LUMINAL – BASAL

A. Data Source:

Human breast cancer cell lines (ETABM157) which have been characterized as luminal or basal were used to develop a signature capable of distinguishing between samples with basal and luminal cell characteristics. Luminal cell lines used were: 600MPE, AU565, BT474, BT483, CAMA1, HCC1428, HCC202, HCC2185, LY2, MCF7, MDAMB134VI, MDAMB175VII, MDAMB361, MDAMB415, MDAMB453, SKBR3, SUM185PE, SUM44PE, SUM52PE, T47D, UACC812, ZR751, ZR7530, and ZR75B. The basal breast cancer cell lines used were: BT20, BT549, HBL100, HCC1143, HCC1187, HCC1500, HCC1569, HCC1937, HCC1954, HCC2157, HCC3153, HCC38, HCC70, HS578T, MCF10A, MCF12A, MDAMB157, MDAMB231, MDAMB435, MDAMB436, MDAMB468, SUM1315MO2, SUM149PT, and SUM159PT.

B. Data Preparation:

- RNA was collected from the described experimental conditions and hybridized to Affymetrix U133A arrays.
- The resulting .CEL files were RMA normalized using Affymetrix Expression Console (ver1.0) and the resulting output saved as lum_bas_train_rma.txt.
- In order to enable this file to be utilized by BINREG (ver2), lum_bas_train_rma.txt was opened in Microsoft Excel and the sample labels were changed from luminal to 0 and basal to 1. This file was saved as lum_bas_rma.xlsx.

C. Generation of the LUMINAL - BASAL Signature:

- In MATLAB, the BINREG (ver2.0) GUI window was opened using the command >>binreg.
- In the GUI window, la_rma.xlsx was imported.
- The parameters were set as summarized in Supplemental Table 1.
- BINREG was then run to generate the signature. The statistical methodology used in this analysis is described in part II of the Supplemental Methods section entitled Analysis of Expression Data for Predicting Pathway Activity.
- The LUMINAL - BASAL signature probe list and regression weights are reported by BINREG in the output file entitled genecoefficient.txt and the image intensity display (or heatmap) of these values is reported in output figure 1. The LUMINAL - BASAL signature gene list is reported in Supplemental Table 1 of the manuscript and the LUMINAL - BASAL signature heatmap is depicted in Supplemental Figure 10 of the manuscript.

- The formal leave-one-out cross validation (Supplemental Figure 10) used to examine the stability and predictive capability of the model is reported by BINREG in the file entitled crossvalidation.txt and the corresponding graph is reported in BINREG figure 6.

HER2+ RELATED SUBGROUP 10 – SUBGROUP 7

A. Data Source:

Human breast cancer samples previously categorized as being HER2+ subgroup 7 or HER2+subgroup 10 from our collection of 1,143 breast tumor samples (1) were used as training data to generate a signature able to distinguish between these two HER2+ subgroups. Samples used to define subgroup 7 were: **GSE1456**: GSM107100, GSM 107102, GSM 107193, GSM 107208, GSM 107219; **GSE1561:** GSM26878, GSM 26900, GSM 26903, GSM 26910; **GSE2034:** GSM36814, GSM 36842, GSM 36879, GSM 36886, GSM 36916, GSM 36944, GSM 36953, GSM 36964, GSM36968, GSM 36978, GSM 37023; **GSE3494:** GSM79128, GSM 79138, GSM79143, GSM 79144, GSM 79173, GSM79180, GSM79196, GSM79240, GSM79259, GSM79272, GSM79273, GSM79274, GSM79294, GSM79298, GSM79308, GSM79344, GSM79346; **GSE4922:** GSM119966; **GSE5460:** GSM124994, GSM125002, GSM125013, GSM125037, GSM125059, GSM125109; **GSE5764:** GSM134696, GSM134699, GSM134700; **GSE6596**: GSM152344, GSM152350, GSM152358; and **ETABM-158:** b0551, b0672, s0175. The samples used for subgroup 10 training data were: **GSE1456:** GSM107109, GSM107183, GSM107197, GSM107205, GSM107228; **GSE1561:** GSM26870, GSM26873, GSM26883; **GSE2034:** GSM36780, GSM36781, GSM36829, GSM36833, GSM36834, GSM36851, GSM36852, GSM36872, GSM36877, GSM36898, GSM36904, GSM36920, GSM36975, GSM36992, GSM36995, GSM37001, GSM37016, GSM37020, GSM37031, GSM37034, GSM37036, GSM37042, GSM37044, GSM37049, GSM37054; **GSE3494:** GSM79116, GSM79189, GSM89233, GSM89238, GSM79251, GSM79340, GSM79341, GSM79363; **GSE3744:** GSM85495, GSM85498, GSM85504, GSM85505; **GSE4922:** GSM119938, GSM119954; **GSE5460:** GSM125048, GSM125069, GSM125070, GSM125085, GSM125090, GSM125115, GSM125116; and **ETABM-158:** b0252, b0372, b0664, s0046, s0059.

B. Data Preparation:

- RNA was collected from the described experimental conditions and hybridized to Affymetrix U133A arrays.
- The resulting .CEL files were RMA normalized using Affymetrix Expression Console (ver1.0) and the resulting output saved as her10_her7_train_rma.txt.
- In order to enable this file to be utilized by BINREG (ver2), her10_her7_train_rma.txt was opened in Microsoft Excel and the sample labels were changed from subgroup 10 to 0 and subgroup 7 to 1. This file was saved as her10_her7_rma.xlsx.

C. Generation of the SUBGROUP 10 – SUBGROUP 7 Signature:

- In MATLAB, the BINREG (ver2.0) GUI window was opened using the command >>binreg.
- In the GUI window, la_rma.xlsx was imported.
- The parameters were set as summarized in Supplemental Table 1.
- BINREG was then run to generate the signature. The statistical methodology used in this analysis is described in part II of the Supplemental Methods section entitled Analysis of Expression Data for Predicting Pathway Activity.
- The SUBGROUP 10 – SUBGROUP 7 signature probe list and regression weights are reported by BINREG in the output file entitled genecoefficient.txt and the image intensity display (or heatmap) of these values is reported in output figure 1. The SUBGROUP 10 – SUBGROUP 7signature gene list is reported in Supplemental Table 1 of the manuscript and the SUBGROUP

10 – SUBGROUP 7 signature heatmap is depicted in Supplemental Figure 11A of the manuscript.
- The formal leave-one-out cross validation (Supplemental Figure 11B) used to examine the stability and predictive capability of the model is reported by BINREG in the file entitled crossvalidation.txt and the corresponding graph is reported in BINREG figure 6.

II. Identification of Patterns of Pathway Activity in Human Breast Tumors
This section will summarize the methods used to identify patterns of pathway activity in a meta-analysis of human breast tumor samples including the sources of the breast tumor datasets, as well as the methods used in the normalization of the datasets, the prediction of pathway activity, and identification of patterns of pathway activity.

A. Data Sources:

Two meta-datasets are utilized in this manuscript, both have been previously described in great detail, including the normalization methods [14]. The first (primary dataset) is composed of 1,143 samples from 10 independent datasets: GSE1456, GSE1561, GSE2034, GSE3494, GSE3744, GSE4922, GSE5460, GSE5764, GSE6596, and E-TABM-158. The validation dataset is comprised of 547 tumors from GSE6532 and a second dataset from MDACC. Dataset sample information is summarized in Table S2.

B. Determining Predicted Probability of Pathway Activity in Independent Samples
- In order to determine the predicted pathway activity of breast tumor samples, the collection of 1,143 or 547 breast tumors was merged with the training data for each signature.
- For each of the pathways described in Part I, the training data and tumor dataset (using the appropriate RMA or MAS5log2 version) was merged using FileMerger, as described for the validation of each signature. The resulting file was examined and we verified that the data matrix contained the Affymetrix HU133A probe list and that samples were in the same order as the originating files.
- In order to prepare the file for use in BINREG, each merged (training + tumor) was opened using Microsoft Excel and the sample labels for the training data were changed to 0 and 1 as previously described, the validation sample labels were changed to 2, and the resulting file saved.
- In order to run the pathway analysis, BINREG was opened in MATLAB using the *binreg* command as previously described.
- In the BINREG GUI window, each file was imported and, as before, the pathway-specific parameters reported in Supplemental Table 1 were set.
- As previously described, the predicted probability of pathway activity is reported by BINREG in an output file entitled validation.txt. Column A of this file reports the position of each sample in the input file. Column C reports the average predicted probability for each sample.

C. Determining Patterns of Pathway Activity
- To determined patterns of predicted probabilities of pathway activity (Figure 1A), the matrix of predicted probabilities of pathway activity of the initial dataset was used for hierarchical clustering.
- This file was imported into Cluster3.0.
- Hierarchical clustering using complete linkage clustering with an un-centered correlation matrix was used to cluster genes and arrays.
- The resulting data was visualized using Java TreeView and the gene and array dendrograms were captured.
- In order to generate the image intensity map of clustered probabilities of pathway activity, the matrix of clustered probabilities was loaded into MATLAB using the load function. The heatmap

of predicted probabilities of pathway activity was generated using the imagesc function with the default parameters.
- The heatmap and dendrograms were reassembled into a single figure in Adobe Photoshop; this figure is presented as Figure 1A in the manuscript.
- To quantitatively analyze patterns of pathway activity, a Pearson Correlation was performed.
- The matrix of probabilities of pathway activity was opened in Microsoft Excel.
- The order of the pathways was rearranged by hand to reflect the ordered results of the hierarchical clustering analysis.
- The rearranged data matrix was loaded into MATLAB using the load function and a Pearson Correlation was performed using the Pearson Correlation function.
- The resulting r-values were then imaged using the imagesc function in MATLAB; this image is reported as Figure 1B in the manuscript.

D. Integrating Patterns of Environmental Stress in Breast Tumor Subgroups
- The 17 breast tumor subgroups have been previously described [14].
- In order to integrate the patterns of environmental stress pathway activity into each subgroup, the probabilities of the 22 pathways were reordered by hand based on the pattern established by hierarchical clustering.
- Tumor samples were then arranged by assigned subgroup based on probabilities published in Gatza *et al*. [14].
- Finally heatmaps of each subgroup, depicted in Supplementary Figure 2, were generated using the imagesc function in MATLAB.

III. Analysis of patterns of gene expression in Her2+ related subgroups 7 and 10.

In order to perform gene by gene analyses of patterns of gene expression, RMA normalized Affymetrix U133A data from samples described in Table S2 were compared. To examine expression level differences between normal and tumor tissue, the Affymetrix U133A probe set annotation file (release 28) was acquired from the Affymetrix website and probe sets identified for each gene of interest; in instances where multiple probe sets were identified for a given gene, the average expression of all probe sets was used. The probe sets for each gene, and collections of genes, used in our analyses is reported below:

**Gene of Interest**	**Affymetrix U133A probe sets**
Hypoxia- induced genes	202219_at<br>213843_x_at<br>210854_x_at<br>91684_g_at<br>58696_at<br>218695_at<br>219862_s_at<br>217685_at<br>214301_s_at<br>205670_at<br>214797_s_at<br>218141_at<br>211919_s_at

	209201_x_at<br>217028_at<br>217691_x_at<br>212291_at<br>212634_at<br>212368_at<br>212366_at<br>202972_s_at<br>217047_s_at<br>202973_x_at<br>211126_s_at<br>207030_s_at<br>211935_at<br>202934_at<br>210004_at<br>217356_s_at<br>200650_s_at<br>212143_s_at<br>210095_s_at<br>209732_at<br>202628_s_at<br>202627_s_at<br>202498_s_at<br>202499_s_at<br>202497_x_at<br>203603_s_at<br>210139_s_at<br>212097_at<br>203065_s_at<br>221530_s_at<br>209183_s_at<br>209182_s_at<br>204298_s_at<br>202620_s_at<br>202619_s_at<br>202464_s_at<br>221563_at<br>215501_s_at<br>212689_s_at<br>212293_at<br>212740_at<br>209566_at<br>209421_at

	200632_s_at<br>221009_s_at<br>219232_s_at<br>202733_at<br>212633_at<br>205492_s_at<br>91682_at<br>205493_s_at<br>201249_at<br>205076_s_at<br>202856_s_at<br>202855_s_at<br>201250_s_at<br>201037_at<br>218498_s_at<br>208308_s_at<br>219622_at<br>202887_s_at<br>213640_s_at<br>217383_at<br>202460_s_at<br>202459_s_at<br>205621_at<br>213522_s_at<br>206686_at<br>205199_at<br>208286_x_at<br>218602_s_at<br>210848_at<br>216752_at<br>216436_at<br>222305_at<br>200884_at
TXNIP	201008_s_at<br>201009_s_at<br>201010_s_at
VEGF-A	210512_s_at<br>210513_s_at<br>211527_x_at<br>212171_x_at
EGLN3	219232_s_at
GLUT1	201249_at<br>201250_s_at
DEC1	201169_s_at

	201170_s_at
CA9	205199_at
HIF1α	200989_at
HIF2α	200878_at
ARNT	210828_s_at<br>218221_at<br>218222_x_at
EP300	202221_s_at<br>213579_s_at
JUN	213281_at<br>201464_x_at<br>201465_s_at<br>201466_s_at
AKT3	212609_s_at<br>219393_s_at<br>212607_at
EGFR	201983_s_at<br>210984_s_at<br>211550_at<br>211551_at<br>211607_x_at<br>210984_x_at
cMET	230510_at<br>211599_x_at<br>213807_x_at<br>213816_s_at
CD44	209835_x_at<br>210913_s_at<br>212014_x_at<br>212063_at<br>216056_at<br>217523_at<br>204489_s_at<br>204490_s_at
IGF1R	203627_at<br>203628_at<br>208441_at
LDHA	200650_s_at
GATA3	209602_s_at<br>209603_at<br>209604_at
Basal-Luminal genes	211302_s_at<br>203708_at<br>206538_at<br>205030_at<br>205029_s_at<br>213060_s_at<br>205334_at

	203021_at<br>206953_s_at<br>209465_x_at<br>211737_x_at<br>209466_x_at<br>204636_at<br>204455_at<br>203951_at<br>203881_s_at<br>211466_at<br>213033_s_at<br>213032_at<br>213029_at<br>209290_s_at<br>209289_at<br>211467_s_at<br>209283_at<br>203256_at<br>211002_s_at<br>202504_at<br>205157_s_at<br>201820_at<br>823_at<br>203687_at<br>209293_x_at<br>209291_at<br>201984_s_at<br>201983_s_at<br>202267_at<br>219534_x_at<br>213182_x_at<br>202966_at<br>202965_s_at<br>204437_s_at<br>215034_s_at<br>209386_at<br>215033_at<br>202936_s_at<br>202935_s_at<br>203038_at<br>204124_at<br>203397_s_at<br>204990_s_at

	204989_s_at<br>217901_at<br>221505_at<br>208103_s_at<br>203287_at
KRT5	201820_at
KRT17	205157_s_at<br>212236_x_at
KRT18	201596_x_at
KRT19	201650_at
ERBB2	210930_s_at<br>216836_s_at
